# *Listeria*-vectored cervical cancer vaccine candidate strains reduce MDSCs via the JAK-STAT signaling pathway

**DOI:** 10.1186/s12915-024-01876-3

**Published:** 2024-04-19

**Authors:** Yunwen Zhang, Yao Lei, Qian Ou, Mengdie Chen, Sicheng Tian, Jing Tang, Ruidan Li, Qian Liang, Zhaobin Chen, Chuan Wang

**Affiliations:** 1 West China School of Public Health and West China Fourth Hospital, Sichuan University, Chengdu, China; 2 Shen Zhen Biomed Alliance Biotech Group Co., Ltd, Shenzhen, China

**Keywords:** *Listeria*-vectored tumor vaccines, Cervical cancer, Immunosuppression, MDSCs, JAK-STAT

## Abstract

**Background:**

Immunosuppressive status is prevalent in cancer patients and increases the complexity of tumor immunotherapy. It has been found that *Listeria*-vectored tumor vaccines had the potential ability of two-side regulatory effect on the immune response during immunotherapy.

**Results:**

The results show that the combined immunotherapy with the LM∆E6E7 and LI∆E6E7, the two cervical cancer vaccine candidate strains constructed by our lab, improves the antitumor immune response and inhibits the suppressive immune response in tumor-bearing mice in vivo, confirming the two-sided regulatory ability of the immune response caused by *Listeria*-vectored tumor vaccines. The immunotherapy reduces the expression level of myeloid-derived suppressor cells (MDSCs)-inducing factors and then inhibits the phosphorylation level of STAT3 protein, the regulatory factor of MDSCs differentiation, to reduce the MDSCs formation ability. Moreover, vaccines reduce the expression of functional molecules associated with MDSCs may by inhibiting the phosphorylation level of the JAK1-STAT1 and JAK2-STAT3 pathways in tumor tissues to attenuate the immunosuppressive function of MDSCs.

**Conclusions:**

Immunotherapy with *Listeria*-vectored cervical cancer vaccines significantly reduces the level and function of MDSCs in vivo, which is the key point to the destruction of immunosuppression. The study for the first to elucidate the mechanism of breaking the immunosuppression.

**Supplementary Information:**

The online version contains supplementary material available at 10.1186/s12915-024-01876-3.

## Background

Cervical cancer is a serious threat to women’s health, and the number of new cases and deaths from cervical cancer has been increasing globally over the past 30 years [1]. According to the global cancer burden assessment report released by the International Agency for Research on Cancer, the number of new cases of cervical cancer was 600,000 in 2020, ranking fourth among female cancer diseases. Cervical cancer has become the leading cause of cancer death in women in low- and middle-income countries and regions [2]. High-risk human papillomavirus (HPV) types 16 and 18 are the main causative factors of cervical cancer, and persistent infection with HPV-16 and/or HPV-18 causes approximately 70% of cervical cancer cases. In patients with grade 3 cervical intraepithelial neoplasia (CIN) and invasive cervical cancer, the positive rate of HPV infection has reached 90% [3].

From the beginning of HPV infection until the formation of lesions, viral particles actively circumvent immune surveillance, allowing HPV to persist in the body and gradually induce the immune status of patients to shift to immunosuppression. In the early stage of lesions, HPV induces the immune response to shift toward Th2. In advanced lesions, the immune response capacity is impaired and the response becomes ineffective [4]. Although specific T cells can be detected in tumor tissues and draining lymph nodes of patients with cervical cancer, they are not functionally active. In tumor tissues, an immune molecule expression shift occurs, thus leading to immunosuppression enhancement [5, 6]. The immunosuppressive status is prevalent in cervical cancer patients, which weakens the efficacy of cancer therapy. Therefore, reducing the level of suppressed immune response before or during treatment can facilitate the immune response to shift to an antitumor response dominated by T cells, thus improving the therapeutic effect.

*Listeria monocytogenes* (LM) has been widely used as a vector in research on tumor vaccines for cervical cancer, pancreatic cancer, and prostate cancer [7–9]. Clinical data in recent years have shown that LM-vectored tumor vaccines can significantly improve the antitumor immune response in tumor patients. Their clinical therapeutic value has been demonstrated. Our lab has obtained two cervical cancer vaccine candidate strains, LM∆E6E7 and LI∆E6E7, constructed utilizing attenuated LM (LM∆), which knocked out the virulence genes *actA* and *plcB*, and attenuated *Listeria ivanovii* (LI∆), which knocked out the virulence genes *actA* and *plcB*, as vectors [10, 11]. They express HPV-16 E6 and E7 fusion antigen proteins [12]. Animal experiments confirmed that these two candidate strains could induce specific T cells responses against antigen, and combination immunotherapy further effectively inhibited tumors [12]. Furthermore, we noted that during treatment, combination immunotherapy with LM∆E6E7 and LI∆E6E7 reduced the proportion of regulatory T cells (Treg) infiltrating the tumor sites [12]. Wallecha A et al. also reported that LM-listeriolysin O (LLO)-vectored tumor vaccines decreased the proportions of Treg and myeloid-derived suppressor cells (MDSCs) at the tumor sites in mice with breast and prostate cancer, and this phenomenon was independent of tumor type [13]. The increase in Treg and MDSCs is a distinct characteristic of enhanced immunosuppression. The enrichment of Treg and MDSCs at tumor sites is associated with poor prognosis in tumor patients, and a decrease in the proportions of Treg and MDSCs serves to increase antitumor immunity [14–17]. These studies suggested that the two-sided regulatory effect, that is, to incite an antitumor immune response and break immunosuppression, may be the antitumor mechanism of *Listeria*-vectored tumor vaccines.

With regard to tumor vaccines, most of the existing studies have focused on their therapeutic effects and few have reported their antitumor mechanisms. We have noticed that *Listeria*-vectored tumor vaccines can reduce the suppressive immune response in vivo, which has a positive effect on tumor treatment. Unfortunately, the specific molecular mechanisms and regulatory pathways involved in breaking immunosuppression by *Listeria*-vectored tumor vaccines have not been reported to date. This aspect of research is very important for understanding the therapeutic mechanisms of tumor vaccines to develop better tumor vaccines.

Therefore, in this study, a combined immunotherapy strategy containing LM∆E6E7 and LI∆E6E7, the two cervical cancer vaccine candidate strains constructed by our lab, was used as the research model. Beginning from the analysis of the immune status at several important time points after treatment, this study finally clarified that vaccine immunotherapy weakens the suppressive immune response by regulating the JAK-STAT signaling pathway to reduce the level and immunosuppressive function of MDSCs in tumor-bearing mice. The goal of continuous innovation in cancer treatment protocols is to improve their therapeutic effect. This study reveals the specific antitumor mechanism, especially the mechanism of weakening immunosuppression, of *Listeria*-vectored tumor vaccines, which is conducive to the understanding of immune response changes induced by immunotherapy and which will enrich immunotherapy ideas, providing an important basis for the discovery of new immunomodulatory targets or the expansion of combined therapy.

## Results

### Immunotherapy with the LM∆E6E7 and LI∆E6E7 combination significantly inhibited tumor growth and prolonged survival time

**Fig. 1:**
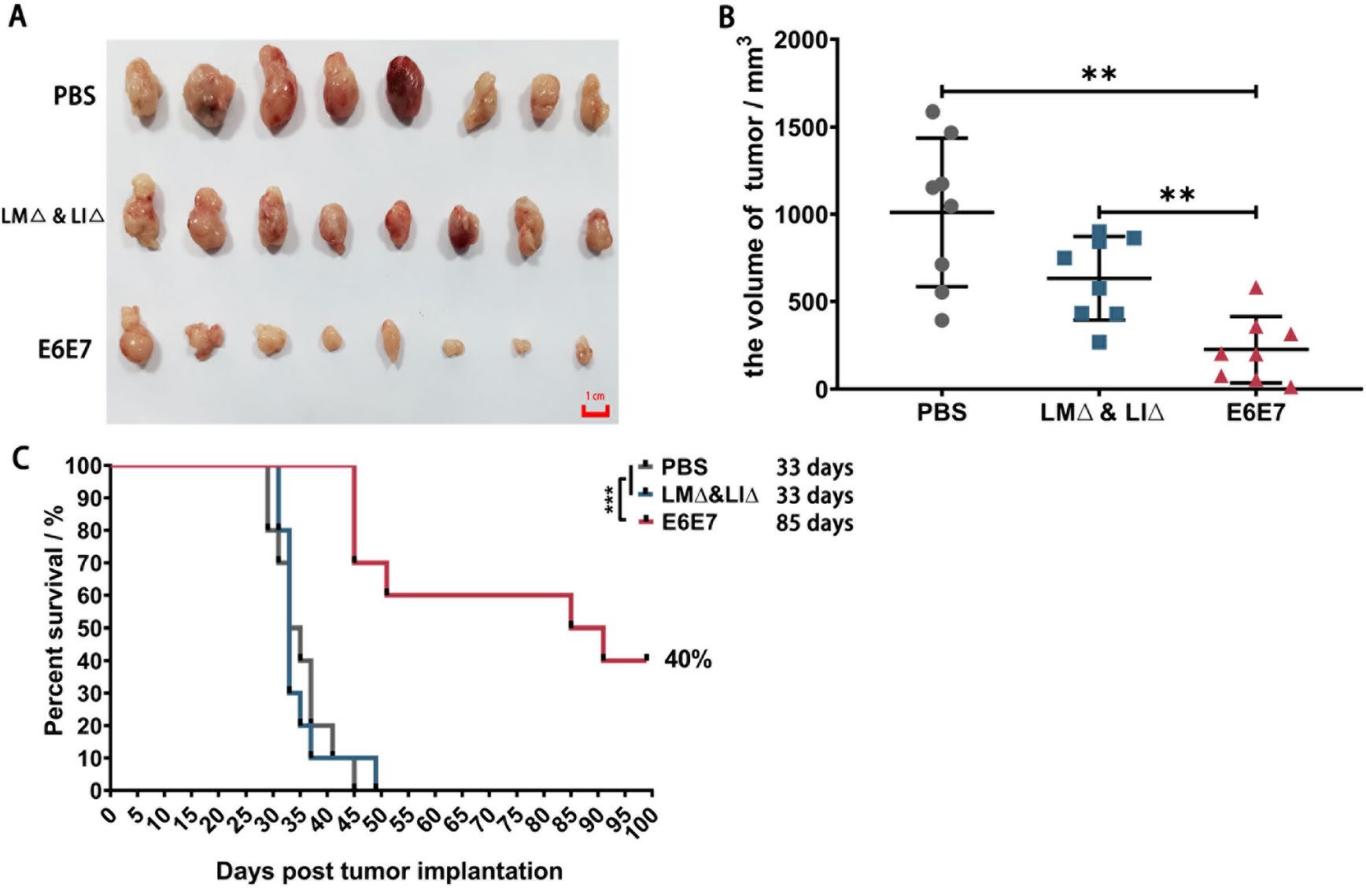
Therapeutic effect of immunotherapy with the LM∆E6E7 and LI∆E6E7 combination on subcutaneous TC-1 cell tumor mouse model. **A** Subcutaneous tumors of tumor-bearing mice taken at 1 week after completion of immunotherapy (*n* = 8). **B** The volume of subcutaneous tumors at 1 week after completion of immunotherapy (*n* = 8). **C** Survival curves in different mice groups, including cure rate and median survival time (*n* = 10)

To observe the treatment effect of immunotherapy, the subcutaneous tumors of mice were isolated 1 week after completion of immunotherapy (28 days). As shown in Fig. 1, the tumor volumes of the E6E7 group (cervical cancer vaccine strain group: tumor-bearing mice were treated with LM∆E6E7 and LI∆E6E7) mice were significantly smaller than those of the PBS group (control group: tumor-bearing mice were treated with PBS solution) and LM∆ and LI∆ group (vaccine vector strain group: tumor-bearing mice were treated with LM∆ and LI∆) (*P* < 0.01, Fig. 1A, B). The observation continued until the 99th day after tumor cell inoculation. The tumor volumes of mice in the PBS and LM∆ and LI∆ groups increased continuously. The mice in both groups died successively due to tumor burden, with a median survival time of 33 days for both groups. However, tumors of 40% mice in the E6E7 group were completely cleared after immunotherapy and without recurrence during the observation. Tumor growth in the remaining 60% of mice was significantly inhibited. Overall, the median survival time of the E6E7 group was 85 days. Immunotherapy with the LM∆E6E7 and LI∆E6E7 combination resulted in a 40% tumor cure rate and significantly prolonged the survival time of the tumor-bearing mice (*P* < 0.001, Fig. 1C).

### Immunotherapy with the LM∆E6E7 and LI∆E6E7 combination disrupted the immunosuppressive status caused by tumor progression

**Fig. 2:**
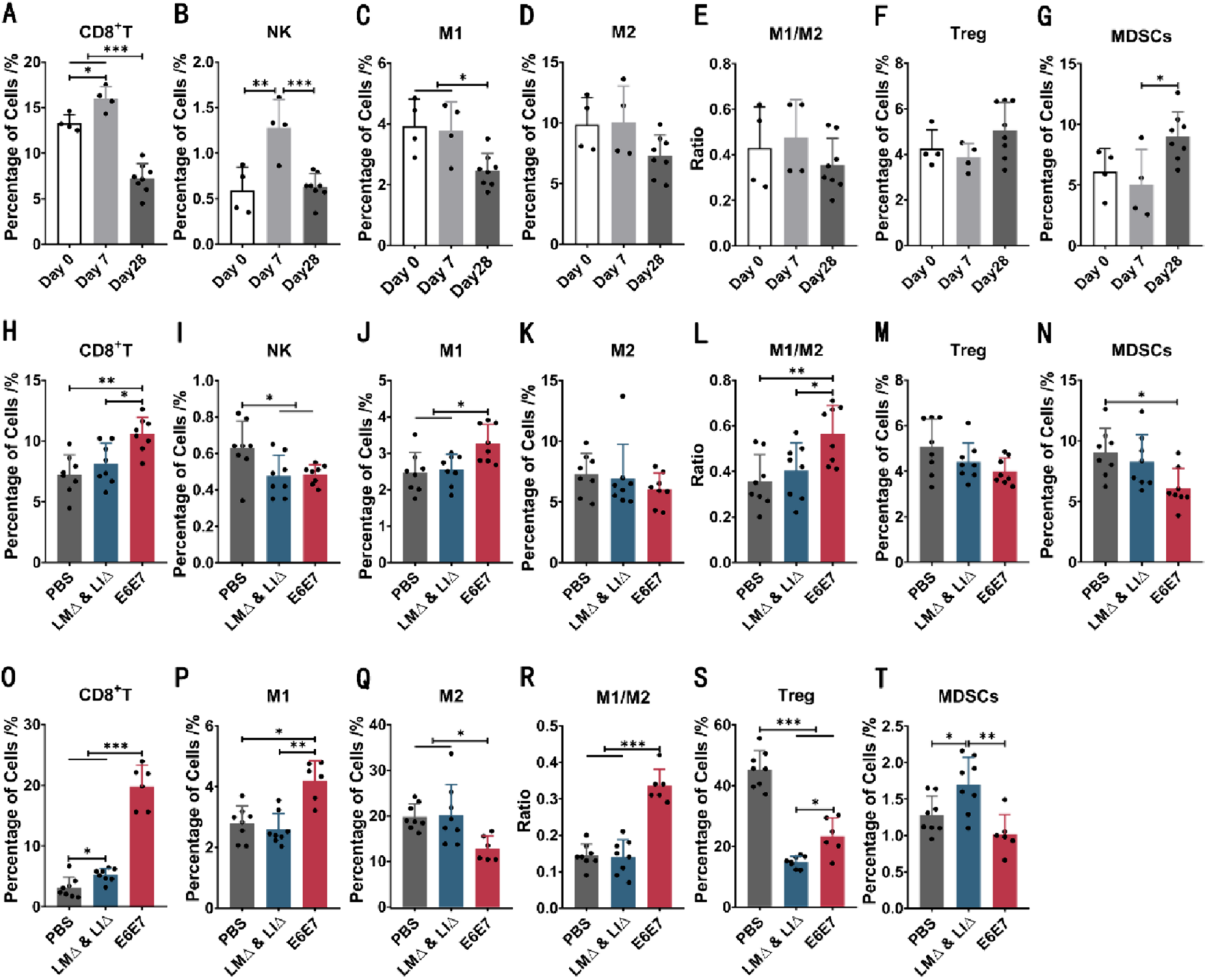
Regulatory effect of immunotherapy with the LM∆E6E7 and LI∆E6E7 combination on the immune status of tumor-bearing mice. Proportions of immune cells in the spleen and TILs of tumor-bearing mice during combination immunotherapy with cervical cancer vaccines. The proportions of CD8^+^ T cells, NK, M1 and M2 types macrophages, Treg, and MDSCs were detected by flow cytometry. **A**–**G** The proportions of immune cells in the spleen of mice without tumor cells inoculation (0 day, *n* = 4) and mice in the PBS group 7 days (*n* = 4) and 28 days (*n* = 8). **H**–**N** The proportions of immune cells in the spleen of tumor-bearing mice 1 week after completion of immunotherapy (28 days). PBS group (*n* = 8), LM∆ and LI∆ group (*n* = 8), E6E7 group (*n* = 8). **O**–**T** The proportions of immune cells in the TILs 1 week after completion of immunotherapy (28 days). PBS group (*n* = 8), LM∆ and LI∆ group (*n* = 8), E6E7 group (*n* = 6). *P < 0.05, **P < 0.01, ***P < 0.001

To understand the evolution of immune status in vivo during tumor progression, the proportions of immune cells in the spleen of the PBS group mice were detected at different timepoints (0 day, 7 days, 28 days). The proportions of CD8^+^ T cells (*P* < 0.05) and natural killer cells (NK) (*P* < 0.01) in tumor-bearing mice at 7 days were significantly increased compared with those in mice not inoculated with tumor cells (0 day). The proportions of other cells did not change significantly (Fig. 2A–G). With tumor progression, the proportions of cells exerting antitumor immune responses, including CD8^+^ T cells (*P* < 0.001), NK cells (*P* < 0.001), and M1-type macrophages (*P* < 0.05), were significantly reduced at 28 days compared with mice at 0 day or 7 days, while the proportion of MDSCs, which promoted tumor progression, was significantly increased (*P* < 0.05), and Treg also showed an increasing trend (*P* > 0.05) (Fig. 2A–G). This indicated that with tumor progression, the level of the antitumor immune response significantly decreased, the level of the suppressive immune response increased simultaneously, and the immune response of tumor-bearing mice was severely imbalanced. However, 1 week after completing the immunotherapy (28 days), compared with the PBS or LM∆ and LI∆ group, the proportions of CD8^+^ T cells (*P* < 0.05) and M1-type macrophages (*P* < 0.05) and the M1/M2 ratio (*P* < 0.05) were significantly increased in the E6E7 group mice (Fig. 2H–L), the proportion of MDSCs was significantly lower (*P* < 0.05), and the proportion of Treg showed a decreasing trend (Fig. 2M, N). At the same time, the immune cell analysis in tumor infiltrating lymphocytes (TILs) (28 days) also showed that compared with the PBS or LM∆ and LI∆ group, the proportions of CD8^+^ T cells (*P* < 0.001) and M1-type macrophages (*P* < 0.05) and the M1/M2 ratio (*P* < 0.001) were significantly higher in the E6E7 group mice, and simultaneously, the proportion of MDSCs (*P* < 0.05) was significantly lower (Fig. 2O–R, T). Moreover, the proportion of Treg (*P* < 0.001) in TILs was significantly lower in the E6E7 and LM∆ and LI∆ groups than in the PBS group (Fig. 2S). It has been found that LM itself is able to reduce the proportion of Treg in tumor-bearing mice in vivo [18]. We also found a reduction in the Treg proportion in the spleen and TILs of LM∆ and LI∆ group mice, consistent with that research. However, we noticed that the significant decrease in the MDSCs proportion in the E6E7 group mice was not associated with the vaccine vector strain efficacy. This result suggests that combined immunotherapy could disrupt the immunosuppressive status formed by tumor progression and further facilitate the antitumor immune response in vivo, in which MDSCs, but not Treg, were the key to disrupting the suppressive immune response.

### Immunotherapy with the LM∆E6E7 and LI∆E6E7 combination induced a high level of antitumor immune response

**Fig. 3:**
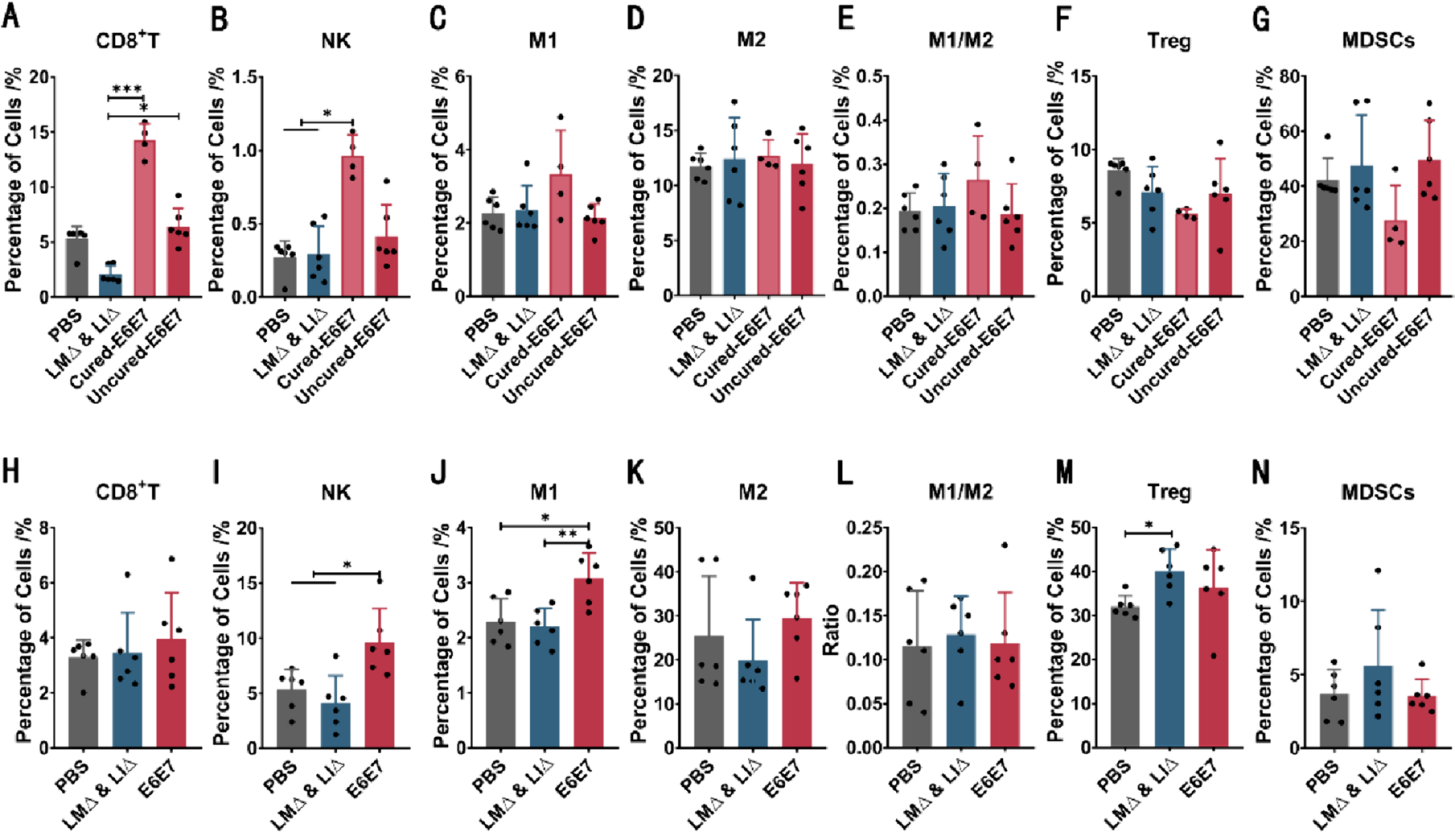
Proportions of immune cells of mice with different outcomes. Tumor-bearing observation endpoint: for tumor-bearing mice, the longest diameter of tumor reaching 20 mm was considered as the endpoint. Cure observation endpoint: for mice free of tumor load, the end of the observation was considered as the endpoint. The proportions of immune cells, including CD8^+^ T cells, NK, M1 and M2 types macrophages, Treg, and MDSCs, were detected by flow cytometry in the spleen and TILs of mice with different outcomes at endpoint. **A**–**G** The proportions of immune cells in the spleen of tumor-bearing mice and cured mice, PBS group (*n* = 6), LM∆ and LI∆ group (*n* = 6), mice cured by cervical cancer vaccines in the E6E7 group (Cured-E6E7, *n* = 4), and mice did not be cured in the E6E7 group (Uncured-E6E7, *n* = 6). **H**–**N** The proportions of immune cells in the TILs of tumor-bearing mice in different groups, PBS group (*n* = 6), LM∆ and LI∆ group (*n* = 6), and E6E7 group (*n* = 6). *P < 0.05, **P < 0.01, ***P < 0.001

Spleens and tumor tissues of mice at the tumor-bearing observation endpoint in each group, as well as spleens of mice at the cure observation endpoint in the E6E7 group, were collected for flow cytometry. For mice cured by the cervical cancer vaccines, the proportions of CD8^+^ T cells (*P* < 0.001) and NK cells (*P* < 0.05) in the spleen were significantly higher than those of tumor-bearing mice in the PBS or the LM∆ and LI∆ group, and the proportion of M1-type macrophages and M1/M2 ratio were also relatively high (*P* > 0.05) (Fig. 3A–E). For the mice that had not been cured in the E6E7 group, the proportion of CD8^+^ T cells in the spleen was also higher than that in mice that died with tumors in the LM∆ and LI∆ group when tested at the observation endpoint (*P* < 0.05) (Fig. 3A). At the observation endpoint, the proportions of NK cells (*P* < 0.05) and M1-type macrophages (*P* < 0.05) in TILs of tumor-bearing mice in the E6E7 group were higher than those of tumor-bearing mice in the PBS and LM∆ and LI∆ groups (Fig. 3I, J). These results suggest that immunotherapy with cervical cancer vaccines significantly induced a high level of antitumor immune response, which was the reason for the delay in tumor progression.

### Analysis of cytokine profiles in serum suggested that the JAK-STAT signaling pathway played an important role in immunotherapy

**Fig. 4:**
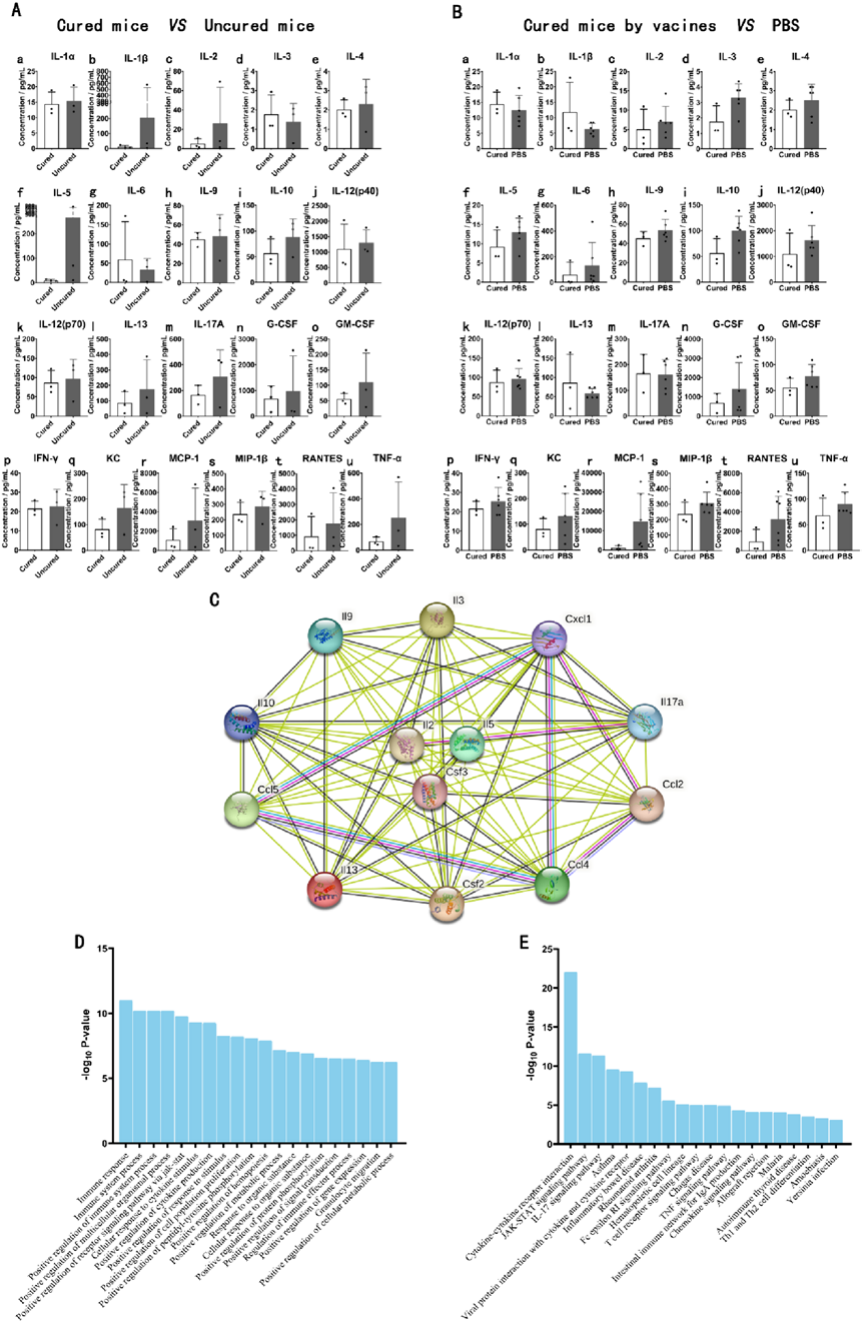
Analysis of cytokine profiles in serum. Determination of the concentrations and analysis of cytokine profiles in serum of mice with different outcomes at endpoint. **A** Comparation of cytokines concentrations of cured mice (Cured, *n* = 3) with uncured mice (Uncured, *n* = 3) in the E6E7 group. **B** Comparation of cytokines concentrations of cured mice in the E6E7 group (Cured, *n* = 3) with tumor-bearing mice (*n* = 6) in the PBS group. **C** Protein–protein interaction (PPI) network diagram. **D** Biological functional GO enrichment analysis. **E** KEGG pathways enrichment analysis

Immunotherapy with the LM∆E6E7 and LI∆E6E7 combination was able to improve the antitumor immune response and reduce the suppressive immune response. The cytokine profiles in endpoint mice with different outcomes were further determined. We found that compared to mice cured by cervical cancer vaccines (Cured) the levels of interleukin-1β (IL-1β), IL-2, IL-5, IL-10, IL-13, IL-17A, keratinocyte-derived chemokine (KC, CXCL1), granulocyte–macrophage colony stimulating factor (GM-CSF), tumor necrosis factor-α (TNF-α), and monocyte chemoattractant protein-1 (MCP-1, CCL2) in the serum of mice that were not cured in the E6E7 group (Uncured) were higher (Fig. 4A). The levels of IL-3, IL-9, IL-10, KC, granulocyte-colony stimulating factor (G-CSF), GM-CSF, KC, MCP-1, macrophage inflammatory protein-1β (MIP-1β, CCL4), interferon-γ (IFN-γ), and regulated on activation in normal T cell expressed and secreted factor (RANTES, CCL5) in the serum of tumor-bearing mice in the PBS group were higher (Fig. 4B). This result suggests that the changes in the levels of these cytokines induced by combined immunotherapy might play an important functional regulatory role in tumor immunotherapy. Bioinformatics analysis was conducted for these cytokines whose content differed between groups by using the STING database, and it was revealed that IL-2, IL-3, IL-5, IL-9, IL-10, IL-13, IL-17A, KC, G-CSF, GM-CSF, MCP-1, MIP-1β, and RANTES formed the major protein–protein interaction network (Fig. 4C). The top 20 most significant items in the GO enrichment analysis were related to the immune response, nonreceptor tyrosine kinase (Janus kinase, JAK)-signal transducer and activator of transcription (STAT) signaling pathway, cell proliferation, tyrosine phosphorylation, etc. The top 20 most significant items in KEGG pathway enrichment analysis included cytokine and receptor interactions, JAK-STAT signaling pathway, IL-17 signaling, T cell receptor signaling, and chemokine signaling pathway. (Fig. 4D, E). Among these, the JAK-STAT signaling pathway plays a key regulatory role.

The JAK-STAT signaling pathway plays an important regulatory role in type I and type II cytokine activities and is involved in a variety of physiological processes such as inflammation, hematopoiesis, immunity, and cell proliferation and apoptosis [19, 20]. The JAK-STAT signaling pathway also plays an important regulatory role in multiple biological processes associated with MDSCs. A variety of inflammatory factors directly induce the differentiation of MDSCs and further regulate the immunosuppressive function of MDSCs via JAK-STAT signaling [21, 22]. Our results on the immune status of mice showed that reducing the proportion of MDSCs in tumor-bearing mice by immunotherapy was the key to disrupting their immunosuppressive status. In summary, these results suggest that combined immunotherapy with LM∆E6E7 and LI∆E6E7 may exert antitumor effects by regulating the level and function of MDSCs through the JAK-STAT signaling pathway.

### Immunotherapy with the LM∆E6E7 and LI∆E6E7 combination inhibited the differentiation and expansion of MDSCs in tumor-bearing mice

**Fig. 5:**
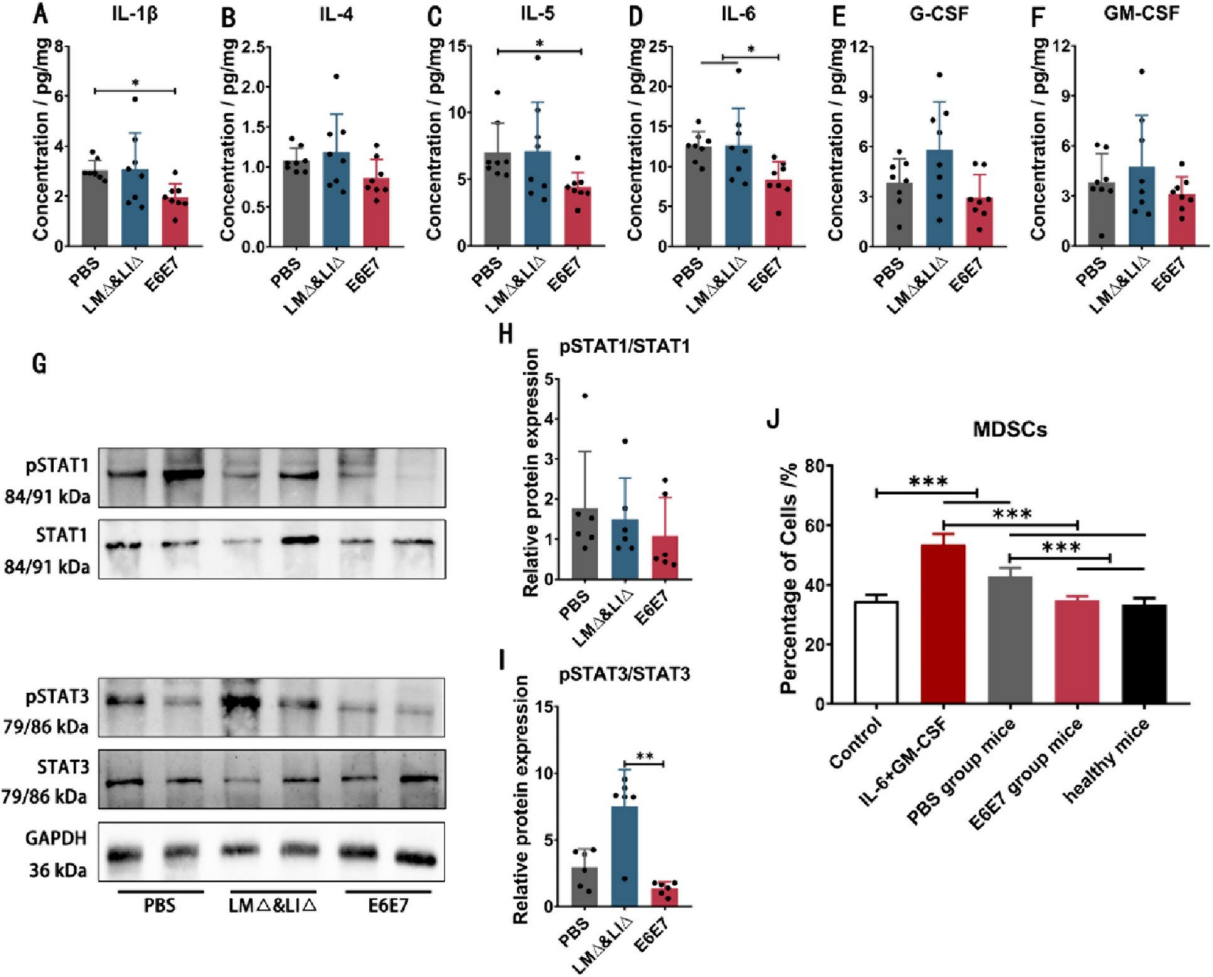
Immunotherapy with the LM∆E6E7 and LI∆E6E7 combination affected the differentiation and expansion of MDSCs in tumor-bearing mice in vivo*.* **A**–**F** Determination of the concentrations of IL-1β, IL-4, IL-5, IL-6, G-CSF, and GM-CSF in spleen tissues by ELISA. PBS group (*n* = 8), LM∆ and LI∆ group (*n* = 8), E6E7 group (*n* = 8). **G**–**I** Determination of the phosphorylation levels of STAT1 and STAT3 protein in spleen tissues by WB. PBS group (*n* = 6), LM∆ and LI∆ group (*n* = 6), E6E7 group (*n* = 6). **J** Flow cytometry to detect the proportions of formed MDSCs in bone marrow cells which were induced by different induction medium (*n* = 6). *P < 0.05, **P < 0.01, ***P < 0.001

During tumor development, abnormally expressed immune molecules act as stimulatory signals to induce the differentiation and expansion of MDSCs in vivo. The proportion of MDSCs in the spleen was significantly reduced in tumor-bearing mice after immunotherapy with the LM∆E6E7 and LI∆E6E7 combination (Fig. 2N). ELISA was performed to determine the expression levels of MDSCs-inducing factors and revealed that at 1 week after completion of immunotherapy (28 days), the concentrations of IL-1β, IL-5, and IL-6 in the spleen of the mice in the E6E7 group were significantly lower (*P* < 0.05) than those in the mice of the PBS group or LM∆ and LI∆ group, and the levels of IL-4, G-CSF, and GM-CSF also showed a decreasing trend (*P* > 0.05) (Fig. 5A–F). These results suggest that immunotherapy reduced the expression level of MDSCs-inducing factors in tumor-bearing mice. MDSCs-inducing factors regulate the differentiation and expansion process of MDSCs mainly by activating STAT1 and STAT3 proteins in the JAK-STAT signaling pathway. At the same time, the western blot (WB) results in spleen tissue protein showed that the phosphorylation level of STAT1 protein was relatively lower (*P* > 0.05) in mice in the E6E7 group compared with that of mice in the PBS group and LM∆ and LI∆ group. Compared with that in the LM∆ and LI∆ group mice, the phosphorylation level of STAT3 protein in the mice in the E6E7 group was significantly lower (*P* < 0.05) (Fig. 5G–I). The bone marrow cells isolated from healthy mice were differentiated into MDSCs under the induction of medium containing mouse spleen grinding supernatant. The results of flow cytometry showed that a certain proportion of MDSCs existed in the bone marrow cells, and the proportion of MDSCs induced by medium containing cytokines IL-6 and GM-CSF was significantly increased (*P* < 0.001). The proportion of MDSCs in the bone marrow cells after induction by medium containing the ground supernatant of the PBS group mouse spleen was significantly increased (*P* < 0.001). In contrast, the proportion of MDSCs in the bone marrow cells after induction by medium containing the grinding supernatant of the E6E7 group mouse spleen was significantly lower than that induced by the grinding supernatant of the PBS group mouse spleen (*P* < 0.001), which was not different from MDSCs induced by the grinding supernatant of healthy mouse spleen and those without induction (*P* > 0.05) (Fig. 5J). These results suggest that the immunosuppressive status induced by tumor progression was conducive to the generation of MDSCs, but immunotherapy with the LM∆E6E7 and LI∆E6E7 combination inhibited this situation by decreasing the expression of MDSCs inducible factors and downregulating the phosphorylation level of STAT3 protein, thus inhibiting the differentiation and expansion of MDSCs in vivo.

### Immunotherapy with the LM∆E6E7 and LI∆E6E7 combination inhibited the enrichment of MDSCs to tumor sites and attenuated MDSCs function by inhibiting activation of the JAK-STAT signaling pathway

**Fig. 6:**
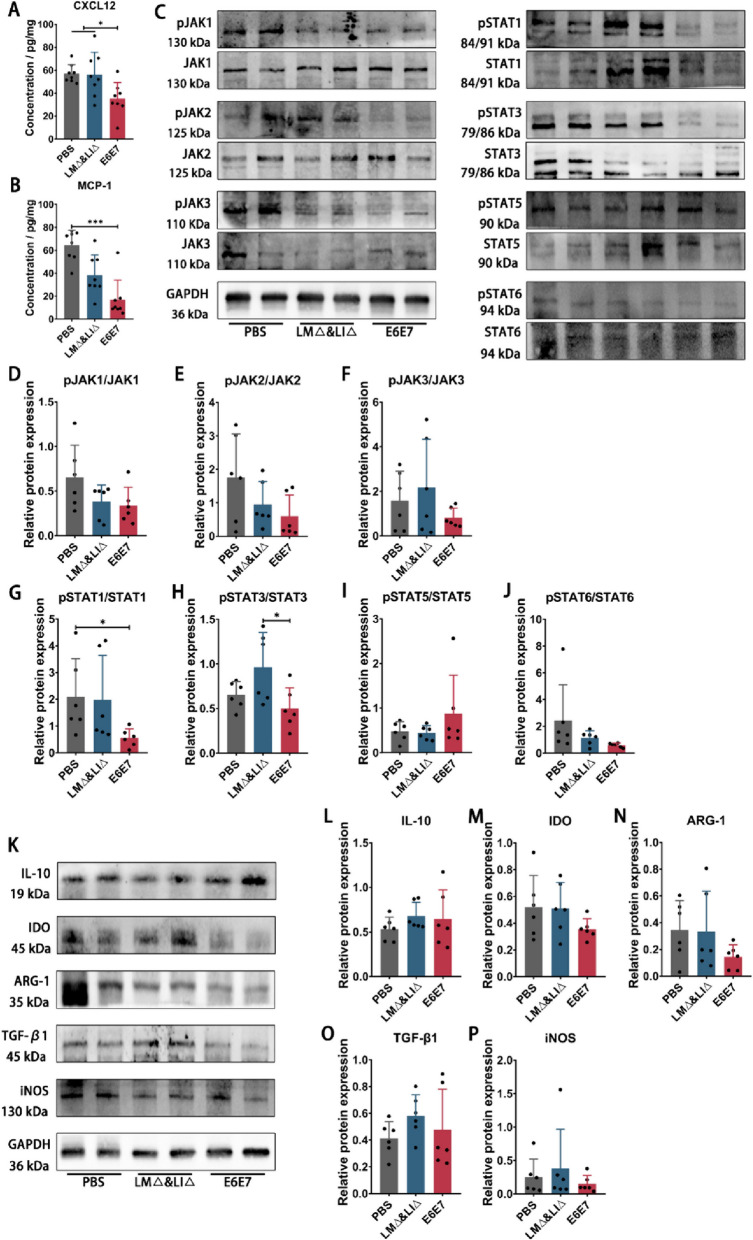
Effect of combined immunotherapy with the LM∆E6E7 and LI∆E6E7 on the enrichment and function of MDSCs at tumor sites. One week after completion of immunotherapy, the concentrations of MDSCs recruitment factors, the phosphorylation levels of the proteins involved in JAK-STAT signaling pathway, and the expression of the molecules related to the immunosuppressive function of MDCSs in tumor tissues were detected. **A**, **B** Determination of the concentrations of CXCL12 and MCP-1 in the spleen of mice by ELISA assay. PBS group (*n* = 8), LM∆ and LI∆ group (*n* = 8), E6E7 group (*n* = 8). **C**–**J** Detection of the phosphorylation levels of JAK1, JAK2, JAK3, STAT1, STAT3, STAT5, and STAT6 proteins in tumor tissues by WB. **K**–**P** Detection of the expression levels of IL-10, IDO, ARG-1, TGF-β1, and iNOS by WB assay. PBS group (*n* = 6), LM∆ and LI∆ group (*n* = 6), E6E7 group (*n* = 6). *P < 0.05, ***P < 0.001

Decreasing the proportion and inhibiting the ability of MDSCs in the tumor microenvironment (TME) are very important for enhancing the antitumor immune response mediated by T cells. We detected a significant reduction in the proportion of MDSCs in TILs after immunotherapy with the LM∆E6E7 and LI∆E6E7 combination in tumor-bearing mice (Fig. 2T). The high expression of MCP-1 and CXCL12 in tumor-bearing mice could enrich MDSCs to the tumor site. ELISA results showed that the expression levels of MCP-1 (*P* < 0.001) and CXCL12 (*P* < 0.05) were significantly lower in the spleen of the E6E7 group mice compared with the PBS or the LM∆ and LI∆ group (Fig. 6A, B). The phosphorylation levels of proteins related to the JAK-STAT signaling pathway in tumor tissues were detected by WB 1 week after completion of immunotherapy. The results showed that the ratios of pJAK1/JAK1, pJAK2/JAK2, and pJAK3/JAK3 in the E6E7 group mice and LM∆ and LI∆ group mice were relatively decreased compared with those in the PBS group mice (*P* > 0.05) (Fig. 6C–F). The ratios of pSTAT1/STAT1 (*P* < 0.05) and pSTAT3/STAT3 (*P* < 0.05) were significantly lower in the E6E7 group mice than in the PBS group or the LM∆ and LI∆ group mice (Fig. 6C, G–J). The expression levels of effector molecules related to MDSCs immunosuppressive function in tumor tissues were determined by WB. The results indicate that, compared with the PBS and LM∆ and LI∆ group mice, the protein expression levels of arginase-1 (ARG-1), indoleamine 2,3-dioxygenase (IDO), and inducible nitric oxide synthase (iNOS) in tumor tissues of the E6E7 group mice showed a decreasing trend (*P* > 0.05), while the protein expression levels of IL-10 and transforming growth factor-β1 (TGF-β1) did not show a notable change (Fig. 6K–P). These results indicate that immunotherapy with the LM∆E6E7 and LI∆E6E7 combination reduced the expression of MDSCs recruitment factors and further inhibited the enrichment and infiltration of MDSCs into tumor sites. Meanwhile, may mainly by inhibiting the phosphorylation levels of the JAK1-STAT1 and JAK2-STAT3 pathways in tumor tissues, the expression of ARG-1, IDO, and iNOS in the TME was decreased, thus further weakening the immunosuppressive function of MDSCs.

## Discussion

While a large number of immune cells infiltrate tumor tissues, the antitumor immune response is extremely low at tumor sites. The immune status of the TME is significantly associated with tumor progression and metastasis. With the continuous malignant transformation of tumor cells, the immune phenotype of tumor cells is reshaped, and tumor cells gain more immune protective mechanisms that promote tumor cell escape from immune surveillance, which is beneficial to the disruption of the antitumor response [23]. The E6 and E7 proteins directly induce cancerous transformation of host cells [24]; therefore, the E6 and E7 proteins have become ideal tumor-associated antigens (TAAs) for tumor vaccines for cervical cancer. We used the E6 and E7 fusion protein of HPV-16 as tumor antigens to develop our cervical cancer vaccines [12]. A clinical study showed that the E7 protein of HPV-16 may induce cross-reaction with proteins expressed by other types of HPV [25]. This suggests that tumor vaccines using the E6 and E7 fusion protein of HPV-16 as antigens may not be limited to providing treatment only for cervical cancer caused by HPV-16 but also for cancer caused by other types of HPV. Regarding vaccine vectors, at present attenuated LM has been widely used in tumor vaccines. LI, another species of the genus *Listeria*, has low pathogenicity to humans, and it expresses ivanolysin O (ILO), which can also assist LI in escaping phagosomes; thus, LI has a similar antigen-presenting ability as LM [26, 27]. Previous results by our team on LI∆-vectored vaccines showed that LI∆-vectored vaccines had good biosafety and immunogenicity [28], confirming that LI∆ has research value and potential application in developing tumor vaccines. We also found that cross-combination immunization with LM∆E6E7 and LI∆E6E7 reduced the anti-carrier effect induced by continuous immunization with the same vaccine and thus induced a more effective antitumor immune response [12]. Therefore, in this study, all experiments were performed based on the combination immunotherapy strategy that contained two cervical cancer vaccine candidate strains, LM∆E6E7 and LI∆E6E7.

Continuous monitoring of the immune status of tumor-bearing mice showed that the suppressive immune response gradually became the dominant immune response as the tumor progressed (Fig. 2A–G), which was consistent with the evolution of the immune status in the bodies of cervical cancer patients [29]. However, combined immunotherapy with LM∆E6E7 and LI∆E6E7 reversed this situation. The immunotherapy improved the antitumor immune response in the spleen and TME of tumor-bearing mice and disrupted the suppressive immune status induced by the tumor (Fig. 2H–T) to achieve the therapeutic effect of inhibiting tumor progression and even completely eliminating the tumor, with a tumor cure rate of 40% in mice (Fig. 1B).

A decrease in NK cells proportion was observed in the spleen of tumor-bearing mice in the E6E7 and LM∆ and LI∆ groups 1 week after completion of immunotherapy (Fig. 2I), but this may be related to an innate immune response against bacterial infection in the body. In a study regarding LM infection, researchers detected the proportion of NK cells in the spleen after intravenous inoculation of LM in mice, and they observed a significant decrease in the proportion of NK cells within six consecutive days after inoculation [30]. In this study, at the endpoint, the level of antitumor immune response in mice cured by immunotherapy was significantly higher than that in mice with tumor burden. Moreover, even the tumor-bearing mice that were not cured by immunotherapy had a higher level of antitumor response than the mice in the PBS and LM∆ and LI∆ groups (Fig. 3). In a clinical study of tumor vaccine treatment for cervical cancer, it was reported that after CIN2/3 patients with HPV-16 or HPV-18 infection received the DNA vaccine VGX-3100, the specific T cell response to E6 and E7 was 9.5-fold higher than that in patients who received placebo. Those who experienced lesion regression and viral clearance also had higher T cell responses than subjects without response [31]. This further indicated that the high level of antitumor immune response induced by immunotherapy with the LM∆E6E7 and LI∆E6E7 combination promoted a favorable prognostic outcome for tumor treatment.

It has been found that LM itself is able to reduce the proportion of Treg in tumor-bearing mice. However, the decrease in the Treg proportion is not due to a reduction in the absolute count of Treg but because of the expansion of Foxp3^−^CD4^+^ T cells induced by LLO [18]. In this study, the proportion of Treg in the spleen and TILs of tumor-bearing mice at 1 week after completion of immunotherapy (28 days) in the LM∆ and LI∆ group was also decreased compared with that in the PBS group (Fig. 2M, S), indicating that the vaccine vector bacteria themselves did affect the proportion of Treg in tumor-bearing mice. This finding also suggests that the reduction in the Treg proportion alone did not play a major role in disrupting the suppressive immune response during combined immunotherapy.

Moreover, we noticed that there was no difference in the proportion of MDSCs in the spleen and TILs in the PBS and LM∆ and LI∆ group mice, but the proportion of MDSCs was significantly lower in the E6E7 group mice (Fig. 2N, T), indicating that the decrease in the proportion of MDSCs was independent of the vaccine vector bacteria. Some studies have reported that attenuated LM can be carried by MDSCs and then enter the tumor sites by utilizing the immunosuppressive status of MDSCs themselves, and then LM activate the immune response to attack tumor cells via the transmission of LM between tumor cells, thus achieving the effect of tumor suppression [32, 33]. It was possible for the cervical cancer vaccine strains and vaccine vector strains to enter the tumor sites through blood flow by intravenous (i.v.) injection. However, we did not observe any difference in tumor growth between the LM∆ and LI∆ groups and the PBS group (Fig. 1), further demonstrating that the tumor suppression effect of the cervical cancer vaccines was irrelevant to the vaccine vector bacteria. Significantly reducing the level of MDSCs in tumor-bearing mice was the key to disrupting the suppressive immune response by immunotherapy with the LM∆E6E7 and LI∆E6E7 combination.

Importantly, MDSCs play a regulatory role in the immunosuppressive network of the TME. In addition to their potent immunosuppressive function in T cell responses, MDSCs can also induce the recruitment and expansion of Treg by secreting ARG and TGF-β and stimulate the differentiation of Th17 cells into Treg [34–36]. CD40 expressed by MDSCs not only induces T cell tolerance but also induces Treg development and accumulation through the CD40/CD40L interaction [37]. In addition to suppressing T cell function, Treg can also modify MDSCs in the TME by increasing the expression of the suppressor molecule B7-H1 and secreting IL-10, thus reinforcing immunosuppression [38]. IL-10 is a transcriptional regulatory factor of IL-12. After coculture of MDSCs with macrophages, the IL-12 expression level of macrophages is downregulated, and macrophages stimulate MDSCs to produce more IL-10, which intensifies M2 polarization [39]. After immunotherapy, the proportion of M2-type macrophages in the spleen and TILs was significantly decreased, while the proportion of M1-type macrophages, the cells that exert antitumor immune responses, was significantly increased (Fig. 2J–L, P–R). This phenomenon may be related to the decrease in MDSCs levels.

The serum cytokine profiling analysis suggests that combined immunotherapy with LM∆E6E7 and LI∆E6E7 may regulate the level and function of MDSCs through the JAK-STAT signaling pathway (Fig. 4). Existing studies have clarified that high expression of the signaling molecules IL-6, GM-CSF, VEGF, and IL-1β could induce the differentiation, formation, and expansion of MDSCs through activation of JAK-STAT signaling in pathological conditions [40, 41]. The ELISA results show that cervical cancer vaccines inhibited the ability to induce the formation of MDSCs in tumor-bearing mice in vivo (Fig. 5) and inhibited the chemotaxis of MDSCs to the tumor site (Fig. 6A, B), which is consistent with the changes in MDSCs levels detected in tumor-bearing mice.

Activation of the JAK-STAT signaling pathway can also modulate the suppressive function of MDSCs. In addition to promoting the expansion of MDSCs, the activation of STAT1 protein induces the expression of Bcl2a1, ARG-1, iNOS, and TGF-β to enhance the survival and immunosuppressive function of MDSCs [42]. Activation of STAT3 upregulates the expression of ARG-1, recombinant S100 calcium binding protein A8/A9 (S100A8/A9), and NADPH oxidase 2 (NOX2). S100A8/A9, as a proinflammatory factor, activates the expression of genes related to angiogenesis and distal metastasis of tumors and participates in the establishment of the tumor metastasis microenvironment [43]. STAT3 also regulates a series of genes related to cell proliferation and upregulates the expression of the pro-survival genes *Bcl-xl*, *Bcl2*, *cMyc*, *Cyclin D1*, *Survivin*, etc., which facilitates the survival of MDSCs [44, 45]. T cell proliferation and efficacy require the uptake of amino acids for energy metabolism. L-arginine is a nonessential amino acid that is metabolized by the ARG and NOS families. The reduction in L-arginine levels in the TME directly leads to the functional impairment of T cells [46]. Both ARG-1 and iNOS secreted by MDSCs are able to metabolize L-arginine. ARG-1 metabolizes L-arginine to produce L-ornithine and urea, and iNOS metabolizes L-arginine to produce L-citrulline and nitric oxide (NO). L-ornithine is a polyamine precursor required for cell proliferation, and NO is able to inhibit the expression of MHC-II molecules. Thus, L-arginine depletion could inhibit T cell proliferation and promote angiogenesis and tumor metastasis [47–49]. Tryptophan is also a nonessential amino acid that is required for NK and T cells metabolism. IDO secreted by MDSCs degrades tryptophan to prevent T cell proliferation, induce T cell incompetence, and induce the differentiation of CD4^+^ T cells into Treg [50]. WB results (Fig. 6C–J) showed that combined immunotherapy with LM∆E6E7 and LI∆E6E7 significantly decreased the phosphorylation levels of the JAK1-STAT1 and JAK2-STAT3 pathways in tumor tissues, and the expression of downstream regulatory genes was also altered (Additional file 1: Table S1, Fig. S1). Moreover, the expression of IDO, ARG-1, and iNOS was decreased in tumor tissues (Fig. 6C–P). These proteins are regulated by JAK-STAT pathway and play an important role in MDSCs exerting the immunosuppressive function. These results suggested that reduced activation level of the JAK-STAT in tumor tissues might inhibit immunosuppressive function of MDSCs. The function of MDSCs is regulated by multiple signals, and subsequent research can consider isolating the MDSCs from TILs for detection, so as to clarify their specific mechanism.

Clinical and preclinical findings have shown that high levels of MDSCs in cervical cancer patients are associated with rapid tumor progression, resistance to radiation therapy, and formation of premetastatic niches [51–53]. In this study, we have demonstrated that immunotherapy with the LM∆E6E7 and LI∆E6E7 combination can inhibit the formation and function of MDSCs. Currently, several tumor vaccines using LM as a vector, including cervical cancer, malignant pleural mesothelioma, and pancreatic cancer, have been used in the clinical trial stage, but there is still a lack of data on the impact of vaccines on MDSCs in patients [8, 54, 55]. Therefore, evaluating the MDSCs in patients in future relevant clinical trials will provide a strong scientific basis for further elaborating the immunomodulatory effects of *Listeria*-vectored tumor vaccines. The TC-1 cells used in this study are lung epithelial cells expressing the *E6* and *E7* of HPV-16. The mice subcutaneous tumor model established with TC-1 cells have been adopted in many cervical cancer-related studies; however, this model is still different from the primary cervical cancer model or orthotopic transplantation model. Therefore, the results reported in this study may still be different from that obtained from cervical cancer patients, and the immunotherapy efficacy and mechanism of the *Listeria* based vaccines in human tumor microenvironment still remain to be evaluated.

## Conclusions

**Fig. 7:**
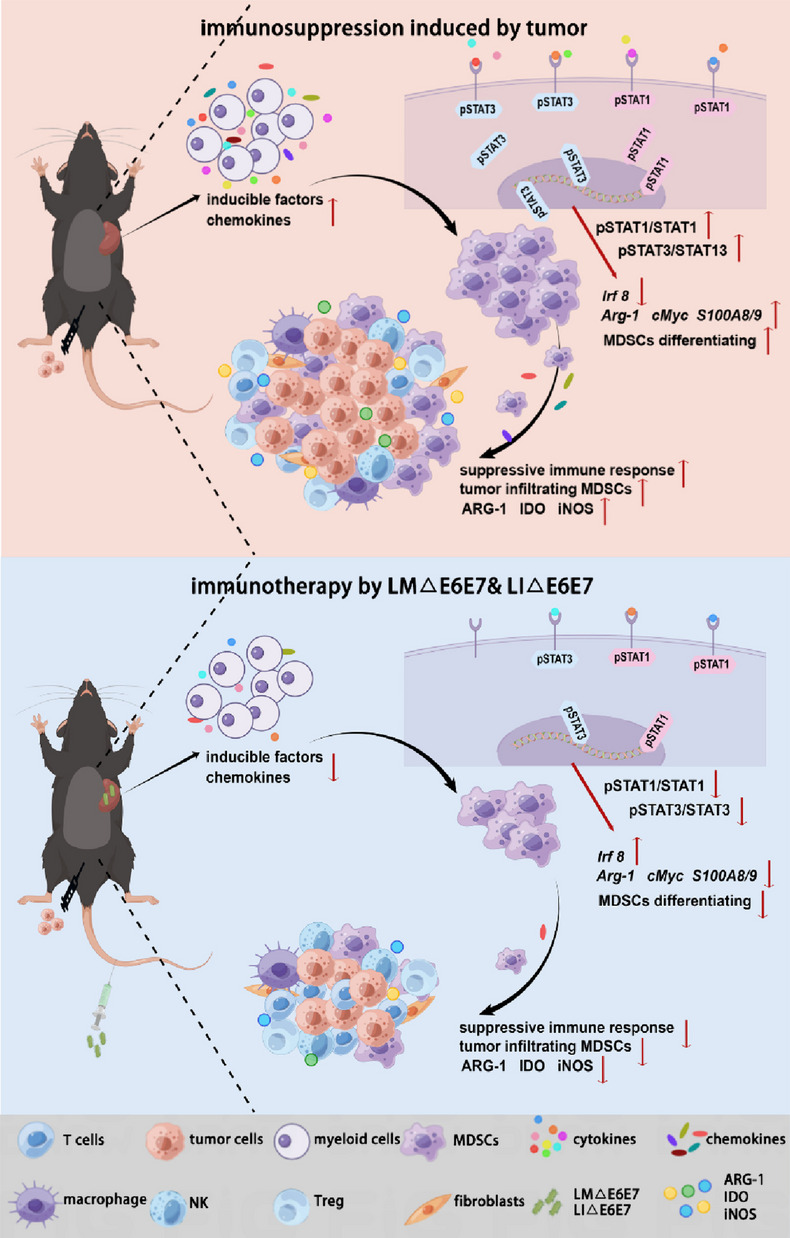
The regulatory mechanism of disrupting the suppressive immune response in vivo by immunotherapy with the LM∆E6E7 and LI∆E6E7 combination in tumor-bearing mice. The figure was drawn on the Figdaw.com website

In summary, our study clarifies that immunotherapy with the LM∆E6E7 and LI∆E6E7 combination could not only improve the antitumor immune response but also reduce the level of the suppressive immune response in tumor-bearing mice. *Listeria*-vectored tumor vaccines exert a two-sided regulatory effect on the immune status in vivo. In this study, it was confirmed for the first time that immunotherapy by using *Listeria*-vectored vaccines could reduce the level and immunosuppressive function of MDSCs via the JAK-STAT signaling pathway to disrupt the suppressive immune response in tumor-bearing mice. The regulatory mechanism is summarized in Fig. 7. This research clearly illustrates the regulatory mechanism by which *Listeria*-vectored tumor vaccines can reverse the immunosuppressive status induced by tumor progression, adding detailed and scientific data about the antitumor mechanism of this kind of tumor vaccine, enriching the basic research data on tumor vaccines, and providing a breakthrough for the in-depth exploration of the tumor suppressive regulatory mechanism. We found that *Listeria*-vectored tumor vaccines have the ability to modulate MDSCs, which may provoke new ideas and references for subsequent combination therapy and provide a valuable reference for basic research on other tumor vaccines.

## Methods

### Mice and cells

Specific pathogen-free (SPF) female C57BL/6 J mice aged 6–8 weeks (Beijing Vital River Laboratory Animal Technology Co., Ltd. China) were housed in the SPF class animal house at the West China School of Public Health, Sichuan University. The animal experimental protocol was approved by the Ethics Committee of the Fourth West China Hospital of Sichuan University/West China School of Public Health (NO. Gwll2022073).

TC-1 cells, derived from lung epithelial cells of C57BL/6 J mice that were co-transformed with genes *E6*, *E7*, and *ras* of HPV-16, were purchased from Beijing Silver Amethyst Biotechnology Co., Ltd. China.

### Therapeutic regimen for tumor-bearing mice

Each mouse was subcutaneously inoculated with 0.1 mL of 1 × 10^6^ cells/mL TC-1 cell suspension in the left abdomen. After 1 week, a rice-like mass could be palpated in the abdomen, indicating that the subcutaneous tumor-bearing mouse model for cervical cancer was successfully established.

The cervical cancer vaccine candidate strains LM∆E6E7 and LI∆E6E7 and vaccine vector strains LM∆ and LI∆ were cultured in BHI medium (LAND BRIDGE, China, 37 °C, 180 r/min). Each strain was collected at the logarithmic growth stage and prepared according to the LD_50_ determined by our laboratory (Additional file 1: Table S2) to the corresponding concentration with sterile PBS (Solarbio, China) for inoculation.

**Fig. 8:**
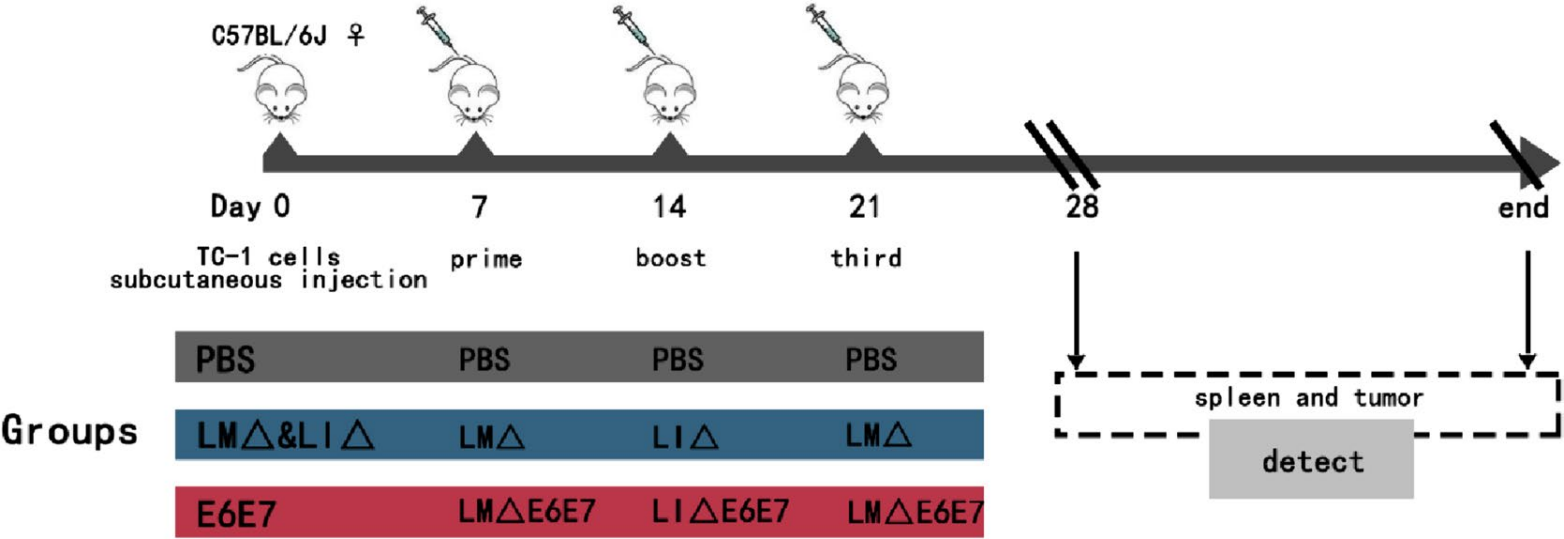
Animal experimental schedule

The therapeutic regimen and experimental schedule for tumor-bearing mice are shown in Fig. 8. The established tumor-bearing mice were randomly divided into three groups: PBS group (*n* = 22), vaccine vector strain group (LM∆ and LI∆, *n* = 18), and cervical cancer vaccine strain group (E6E7, *n* = 18). The day when the tumor cells were subcutaneously inoculated was labeled the 0th day. On the 7th, 14th, and 21th day, tumor-bearing mice in each group were injected intravenously (i.v.) with 100 μL of the corresponding strains or PBS. The longest and shortest diameters of the subcutaneous tumors were measured every 2 days using vernier calipers. When the longest diameter of the tumors reached 20 mm, the tumor-bearing mice were euthanized. This outcome was defined as the “tumor-bearing observation endpoint.” For mice free of tumor load, humanitarian death should be performed at the end of the observation period. Such an outcome was defined as the “cure observation endpoint.” The spleens of mice (0 day, *n* = 4) and tumor-bearing mice (7 days, *n* = 4) without immunotherapy and the spleen and tumor tissues of each group of mice (28 days, *n* = 8) were collected. The sera, spleen, and tumor tissues of each mouse at the observation endpoint were also collected.

### Detection of immune cells in the spleen by flow cytometry

Mouse spleens were ground and filtered through 200-mesh (75 μm) sieves, and the spleen cell suspension was obtained after adding erythrocyte lysate (an aqueous solution containing 0.0026 g/mL Tris and 0.0071 g/mL NH_4_Cl, pH = 7.2–7.4), incubating for 5 min at room temperature and then washing twice with RPMI 1640 medium (Hyclone, USA) Then, cell surface antibodies were added to the spleen cell suspension and incubated at 4 °C for 30 min. After that, the cells were permeabilized with Cytofix/Cytoperm (BD PharMingen, USA) or Fixation/Permeabilization (eBioscience, USA), and blocking buffer was added and incubated for 15 min at 4 °C. Intracellular or intranuclear antibodies were added and incubated at 4 °C for 45 min. After incubation, the cells were washed and resuspended in cell fixative buffer (BD PharMingen, USA) and detected using flow cytometry with NovoCyte (Agilent, USA). Information on the antibodies is shown in Additional file 1: Table S3, and the gating strategy for the flow cytometric analysis of immune cells in the spleen is shown in Additional file 1: Fig. S2.

### Detection of immune cells in the tumor tissue by flow cytometry

After the tumor-bearing mice were sacrificed, subcutaneous tumors were isolated completely. Weigh 0.5 g of tumor tissues were cut and then added to 3 mL of prewarmed (37 °C) 1640 medium containing 5% FBS (Gibco, USA), 1 mg/mL collagenase I (Biofroxx, Germany), and 0.02 mg/mL DNase I (TIANGEN, China). Tumor tissues were digested for 35 min in cell incubators (Thermo Scientific, USA, 37 °C, 5% CO_2_). The digested tissues were ground and filtered through 70-μm cell filters. A Mouse Tumor Infiltrating Lymphocyte Isolation Kit (Solarbio, China) was used to obtain TILs. Cell staining was performed as described above. The gating strategy for the flow cytometric analysis of immune cells in tumor tissue is shown in Additional file 1: Fig. S3.

### Detection of cytokine profiles in serum

The serum was prepared according to the instructions of Bio-Plex Pro Mouse Cytokine Grp I (Bio-Rad, USA), and the cytokine concentration was measured using the Bio-Plex MAGPIX System (Bio-Rad, USA).

### Bioinformatics analysis of cytokine profiles

The protein interaction analysis and enrichment analysis of GO and KEGG pathways of cytokines were performed in the STRING database (https://cn.string-db.org/).

### ELISA

Spleen tissue (20 mg) was homogenized (40 m/s, 20 s, twice) using a homogenizer (MP Biomedicals, USA). Then, the samples were centrifuged at 13,000 g and 4 °C for 10 min, and the supernatant was collected. The concentrations of cytokines and chemokines in spleen grinding supernatant were determined according to the instructions of an ELISA kit (Elabscience, China).

### Western blot

RIPA lysis buffer (Solarbio, China) containing protease inhibitor cocktail (AbMole, USA) and phosphatase inhibitor cocktail (AbMole, USA) was added to 30 mg of tissue, and then the tissue was ground (40 m/s, 20 s, twice) using a homogenizer (MP Biomedicals, USA). The homogenate was incubated at 4 °C for 30 min and then centrifuged at 16,000 g at 4 °C for 10 min. The supernatant was the tissue protein sample. The protein concentration of the tissue protein sample was determined using a BCA Protein Assay Kit (Solarbio, China), and the protein concentration was adjusted to 4 mg/mL by adding loading buffer (NCM Biotech, China). The protein sample was denatured by incubation at 100 °C for 10 min, and WB was performed to detect the expression of proteins in tumor tissue. Information on the antibodies used in WB experiments is shown in Additional file 1: Table S4.

### Isolation of mouse bone marrow cells

Healthy mice were sacrificed and immersed in 75% ethanol solution for 5 min to disinfect, and then the femurs and tibias of the mice were removed completely. Under sterile conditions, the cells in bone tissue were flushed out with RPMI 1640 medium. The cell suspension was filtered through 70-μm cell filters. Then, erythrocyte lysate was added, and the cell suspension was incubated at room temperature for 5 min. The bone marrow cell suspension was obtained after washing with RPMI 1640 medium twice.

### Induction of the differentiation of bone marrow cells by the supernatant of mouse spleen tissue grinding

Spleen tissue (20 mg) was collected from healthy mice and mice from the PBS group and E6E7 group 1 week after completion of immunotherapy. Then, 400 μL of RPMI 1640 medium was added and ground (40 m/s, 20 s, twice) using a homogenizer (MP Biomedicals, USA). Then, 600 μL of RPMI 1640 medium was added to the homogenate and centrifuged at 13,000 g and 4 °C for 10 min. The supernatant was collected and filtered through 0.22μm filters to obtain the spleen tissue grinding supernatant. One hundred microliters of bone marrow cells (2 × 10^6^ cells/mL) was added to each well of a 96-well plate (NEST, China), and then 100 μL of induction medium was added. Induction medium 1: 10% FBS-1640 medium, which served as a negative control. Induction medium 2: 10% FBS-1640 medium containing 20 ng/mL mouse IL-6 (Pepro tech, USA) and 20 ng/mL mouse GM-CSF (Pepro tech, USA), which served as a positive control. Induction medium 3: 10% FBS-1640 medium containing 10% spleen tissue grinding supernatant of PBS group mice. Induction medium 4: 10% FBS-1640 medium containing 10% spleen tissue grinding supernatant of E6E7 group mice. Induction medium 5: 10% FBS-1640 medium containing 10% spleen tissue grinding supernatant of healthy mice. The cells were cultured in cell incubators for 4 days and then collected and detected by flow cytometry. The gating strategy for the flow cytometric analysis of MDSCs in bone marrow cells is shown in Supplementary Fig. 4.

### Statistical analysis

Experimental data are shown as the mean ± standard deviation (mean ± SD). The GraphPad 8.0 software was used for plotting, and SPSS 22.0 was used for statistical analysis. Tukey’s method was used when the data met the normal distribution and the variance was equal; otherwise, a nonparametric test was performed. *P* < 0.05 was considered a statistically significant difference. The mechanism diagram was drawn from the website Figdaw.com.

### Supplementary Information

**Additional file 1:** **Table S1** Primer sequence information of genes. **Table S2** The concentration and inoculum volume of each strain. **Table S3** The information of antibody used in flow cytometry. **Table S4** The information of antibody used in WB. **Figure S1** Effect of combined immunotherapy with LM∆E6E7 and LI∆E6E7 on mRNA expression levels of downstream genes of JAK-STAT pathway in mice tumor tissues. **Figure S2** Gating strategy of flow cytometric analysis of immune cells in the spleen of mice. **Figure S3** Gating strategy of flow cytometric analysis of immune cells in the TILs of mice. **Figure S4** Gating strategy of flow cytometric analysis of MDSCs in bone marrow cells of mice. The images of the original, uncropped blots.

## Data Availability

All data generated or analyzed during this study are included in this published article and its supplementary information file. The antibody, primer information, and unprocessed images of WB are included in Additional file 1. Two vaccine strains LM∆E6E7 and LI∆E6E7 are not deposited in a public repository and they are available upon reasonable request from the corresponding author.

## Abbreviations

HPV: High-risk human papillomavirus
CIN: Cervical intraepithelial neoplasia
LM: *Listeria monocytogenes*
LI: *Listeria ivanovii*
Treg: Regulatory T cells
LLO: Listeriolysin O
MDSCs: Myeloid-derived suppressor cells
NK: Natural killer cells
TILs: Tumor infiltrating lymphocytes
IL: Interleukin
KC: Keratinocyte-derived chemokine
GM-CSF: Granulocyte-macrophage colony stimulating factor
TNF-α: Tumor necrosis factor-α
MCP-1: Monocyte chemoattractant protein-1
G-CSF: Granulocyte-colony stimulating factor
MIP-1β: Macrophage inflammatory protein-1β
IFN-γ: Interferon-γ
RANTES: Regulated on activation in normal T cell expressed and secreted factor
JAK: Janus kinase
STAT: Signal transducer and activator of transcription
WB: Western blot
TME: Tumor microenvironment
ARG-1: Arginase-1
IDO: Indoleamine 2,3-dioxygenase
iNOS: Inducible nitric oxide synthase
TGF-β1: Transforming growth factor-β1
TAAs: Tumor-associated antigens
ILO: Ivanolysin O
S100A8/A9: Recombinant S100 calcium binding protein A8/A9
NOX2: NADPH oxidase 2
NO: Nitric oxide
SPF: Specific pathogen-free
