# Supplementary material for: Listeria-vectored cervical cancer vaccine candidate strains reduce MDSCs via the JAK-STAT signaling pathway
Source: BMC Biol. 2024 Apr 19;22:88. doi: 10.1186/s12915-024-01876-3 (PMC11031962; doi:10.1186/s12915-024-01876-3)

*Listeria*-vectored cervical cancer vaccine candidate strains reduce MDSCs via the JAK-STAT signaling pathway

Yunwen Zhang^1,2#^, Yao Lei^1#^, Qian Ou^1#^, Mengdie Chen^2^, Sicheng Tian^1^, Jing Tang^1^, Ruidan Li^1^, Qian Liang^1^, Zhaobin Chen^2^*, Chuan Wang^1^*

*Correspondence: Chuan Wang: [wangchuan@scu.edu.cn](mailto:wangchuan@scu.edu.cn);

Zhaobin Chen: [chenzb.md@vip.163.com](mailto:chenzb.md@vip.163.com)

1 West China School of Public Health and West China Fourth Hospital, Sichuan University, Chengdu, China.

2 Shen Zhen Biomed Alliance Biotech Group Co., Ltd., Shenzhen, China.

^#^ These authors share first authorship.

**Supplementary Table**

**Table S1 Primer sequence information of genes.**

| gene | Forward primers | Reverse primers |
| --- | --- | --- |
| *Gapdh* | ACCCAGAAGACTGTGGATGG | ACACATTGGGGGTAGGAACA |
| *Bcl-xl* | GCCTTTTTCTCCTTTGGCGG | TCCACAAAAGTGTCCCAGCC |
| *Irf8* | GAACCGGCGGCAGGATGT | ATGCTTCCAGGGGATACGGA |
| *Nox2* | AATCCCTGCTCCCACTAACA | TTTCAAGATGCGTGGAAACTAC |
| *cMyc* | TGATGACCGAGTTACTTGGAG | GGCTGGTGCTGTCTTTGC |
| *S100A8* | AAATCACCATGCCCTCTACAAG | CCCACTTTTATCACCATCGCAA |
| *S100A9* | ACCACCATCATCGACACCTTC | AAAGGTTGCCAACTGTGCTTC |
| *iNos* | GTGGTGACAAGCACATTTGG | AAGGCCAAACACAGCATACC |
| *Arg-1* | TTTAGGGTTACGGCCGGTG | TTTGAGAAAGGCGCTCCGAT |

**Table S2 The concentration and inoculum volume of each strain.**

| Strain | LD_50_ | Concentration | Inoculum volume |
| --- | --- | --- | --- |
| LM∆ | 5×10^7^ cfu/mL | 5×10^7^ cfu/mL | 100 μL |
| LI∆ | 2×10^8^ cfu/mL | 2×10^8^ cfu/mL | 100 μL |
| LM∆E6E7 | 1.3×10^8^ cfu/mL | 1.3×10^8^ cfu/mL | 100 μL |
| LI∆E6E7 | 4×10^8^ cfu/mL | 4×10^8^ cfu/mL | 100 μL |

**Table S3 The information of antibody used in flow cytometry.**

| Name | Clone No | Company |
| --- | --- | --- |
| FITC Rat Anti-Mouse CD3 | 17A2 | BD PharMingen, USA |
| FITC Rat Anti-Mouse CD4 | RM4-5 | BD PharMingen, USA |
| PE Rat Anti-Mouse CD8a | 53-6.7 | BD PharMingen, USA |
| FITC Anti-mouse CD11b Antibody | M1/70 | eBioscience, USA |
| PE Anti-mouse F4/80 Antibody | T45-2342 | BD PharMingen, USA |
| PE-Cy7 Anti-mouse CD86 Antibody | GL1 | eBioscience, USA |
| APC Anti-mouse CD206 Antibody | MR6F3 | eBioscience, USA |
| PE Anti-mouse NK1.1 Antibody | PK136 | eBioscience, USA |
| PerCP Cy5.5 Anti-mouse Gr-1 Antibody | R86-BC5 | eBioscience, USA |
| PE Anti-mouse FOXP3 Antibody | FJK-16s | eBioscience, USA |
| APC Anti-mouse CD25 Antibody | PC61.5 | eBioscience, USA |
| Live/Dead™ Fixable Violet Dead Cell Stain Kit | - | Invitrogen, USA |
| Anti-mouse CD32/16 Purified | 93 | eBioscience, USA |

**Table S4 The information of antibody used in WB.**

| Name | Company | Item No | Molecular weight of protein (kDa) |
| --- | --- | --- | --- |
| JAK1 Antibody | Affinity Biosciences, USA | AF5012 | 130 |
| Phospho-JAK1 Antibody | Affinity Biosciences, USA | AF2012 | 130 |
| JAK2 Antibody | Affinity Biosciences, USA | AF6022 | 125 |
| Phospho-JAK2 Antibody | Affinity Biosciences, USA | AF3024 | 125 |
| JAK3 Antibody | Abclonal, China | A0748 | 110 |
| Phospho-JAK3 Antibody | Abclonal, China | AP0532 | 110 |
| STAT1 Antibody | Affinity Biosciences, USA | AF6300 | 84、91 |
| Phospho-STAT1 Antibody | Affinity Biosciences, USA | AF3300 | 84、91 |
| STAT3 Antibody | Affinity Biosciences, USA | AF6294 | 79、86 |
| Phospho-STAT3 Antibody | Affinity Biosciences, USA | AF3293 | 79、86 |
| STAT5 Antibody | Affinity Biosciences, USA | AF6305 | 90 |
| Phospho-STAT5 Antibody | Affinity Biosciences, USA | AF3305 | 90 |
| STAT6 Antibody | Affinity Biosciences, USA | AF6302 | 94 |
| Phospho-STAT6 Antibody | Affinity Biosciences, USA | AF3301 | 94 |
| ARG1 Antibody | Affinity Biosciences, USA | DF6657 | 35 |
| iNOS Antibody | Affinity Biosciences, USA | AF0199 | 130 |
| IL-10 Antibody | Affinity Biosciences, USA | DF6894 | 19 |
| TGF-β1 Antibody | Affinity Biosciences, USA | AF1027 | 45 |
| IDO1 Antibody | Abclonal, China | A1614 | 45 |
| GAPDH Mouse Monoclonal  Antibody | Origene, USA | TA802519 | 36 |
| Goat anti-Mouse IgG (H&L)  （HRP conjugate） | ZENBIO, China | 511103 | - |
| Goat anti-Rabbit IgG (H&L)  （HRP conjugate） | ZENBIO, China | 511203 | - |

**Supplementary Figure**


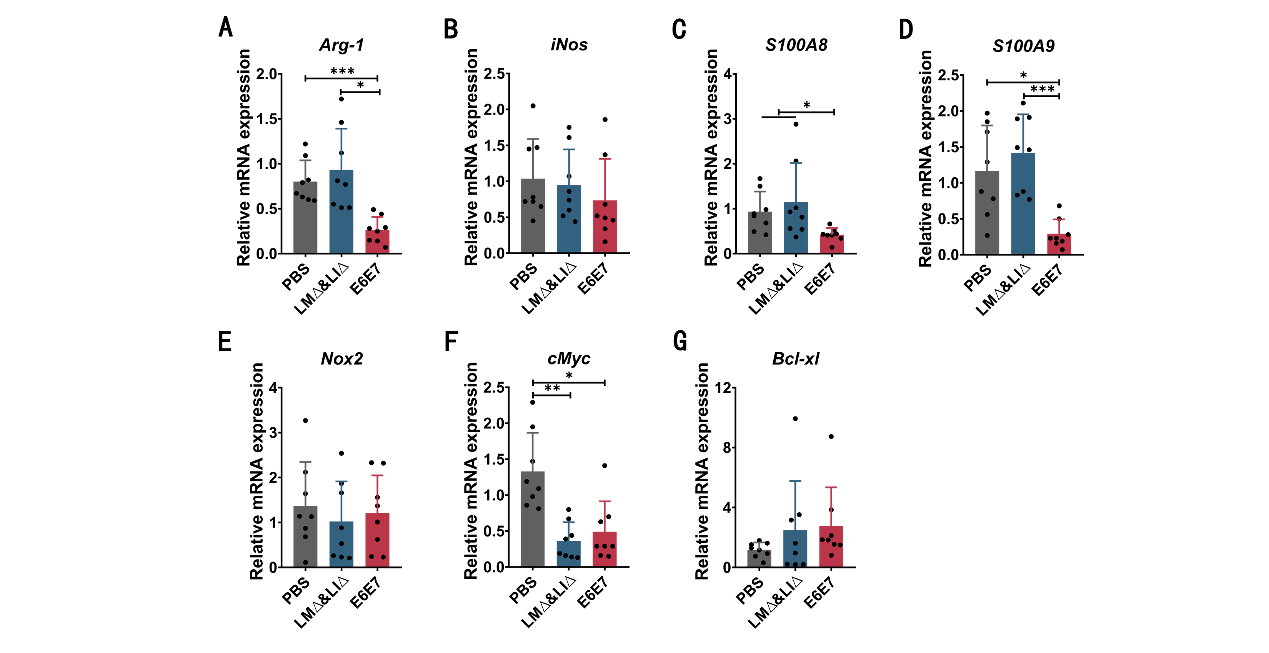


**Fig. S1 Effect of combined immunotherapy with LM∆E6E7 and LI∆E6E7 on mRNA expression levels of downstream genes of JAK-STAT pathway in mice tumor tissues.** RT-qPCR was performed to detect the mRNA expression levels of downstream genes of JAK1-STA1 and JAK2-STAT3 pathway, gene *Arg-1* (A), *iNos* (B), *S100A8* (C), *S100A9* (D), *Nox2* (E), *cMyc* (F) and *Bcl-xl*, in tumor tissues. PBS (n=8), LM∆ & LI∆ (n=8), and E6E7 (n=8). **P*<0.05, ***P*<0.01, ****P*<0.001.


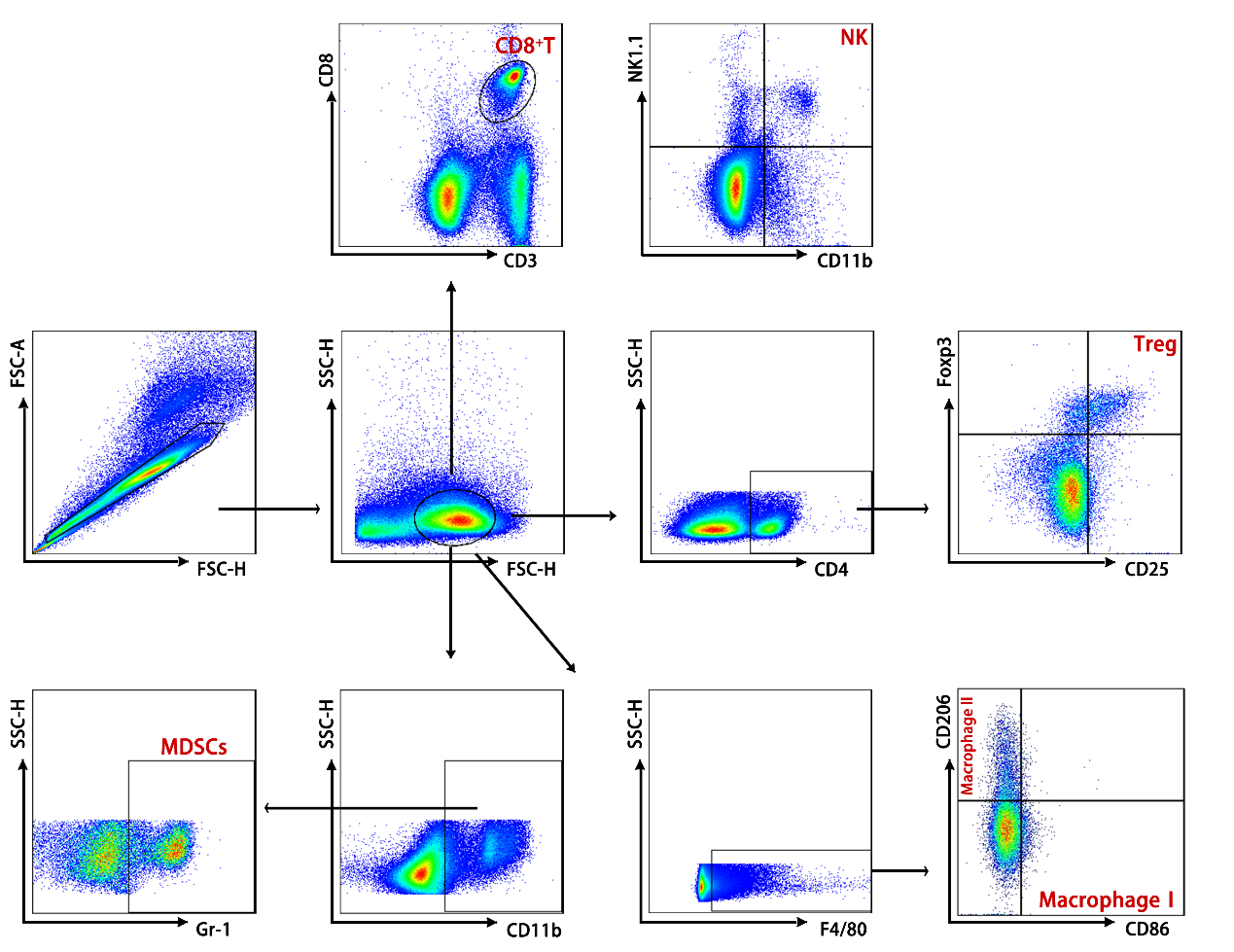


**Fig. S2 Gating strategy of flow cytometric analysis of immune cells in the spleen of mice.**


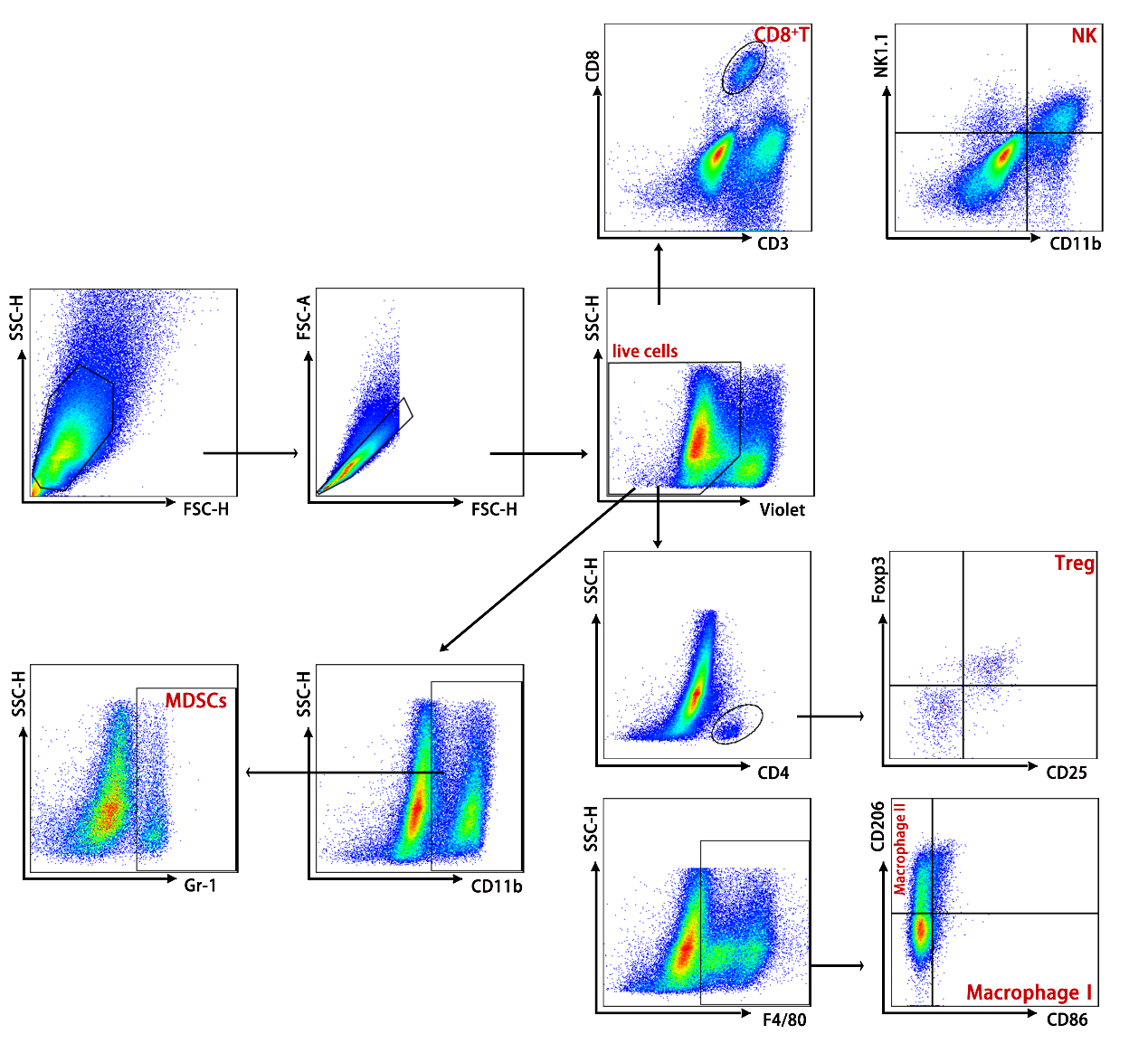


**Fig. S3 Gating strategy of flow cytometric analysis of immune cells in the TILs of mice.**


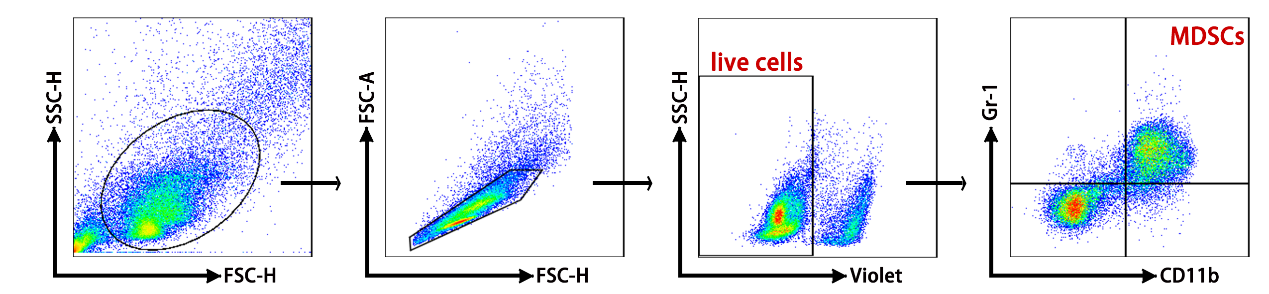


**Fig. S4 Gating strategy of flow cytometric analysis of MDSCs in bone marrow cells of mice.**

**unprocessed images of WB in Fig 5**

Order:

| PBS | PBS | LM∆ & LI∆ | LM∆ & LI∆ | E6E7 | E6E7 | Others | Others |
| --- | --- | --- | --- | --- | --- | --- | --- |

pSTAT1


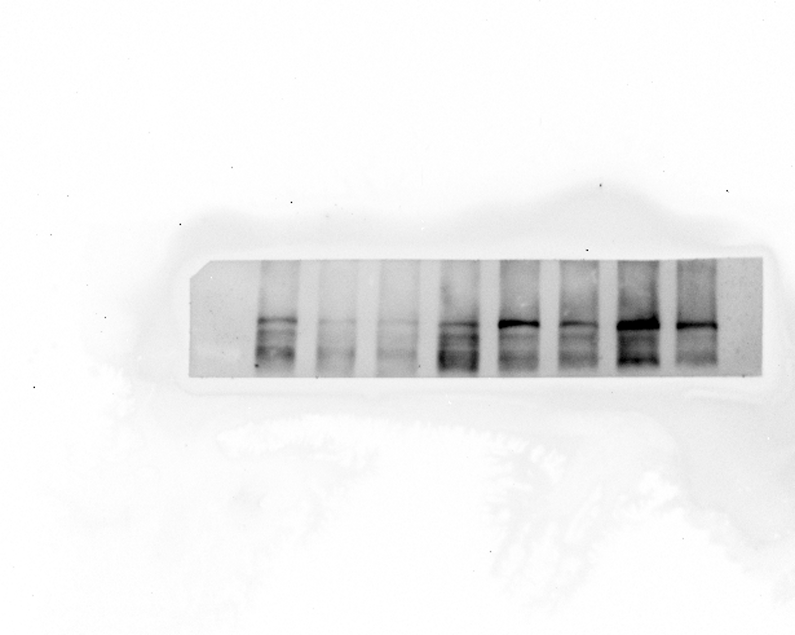


STAT1


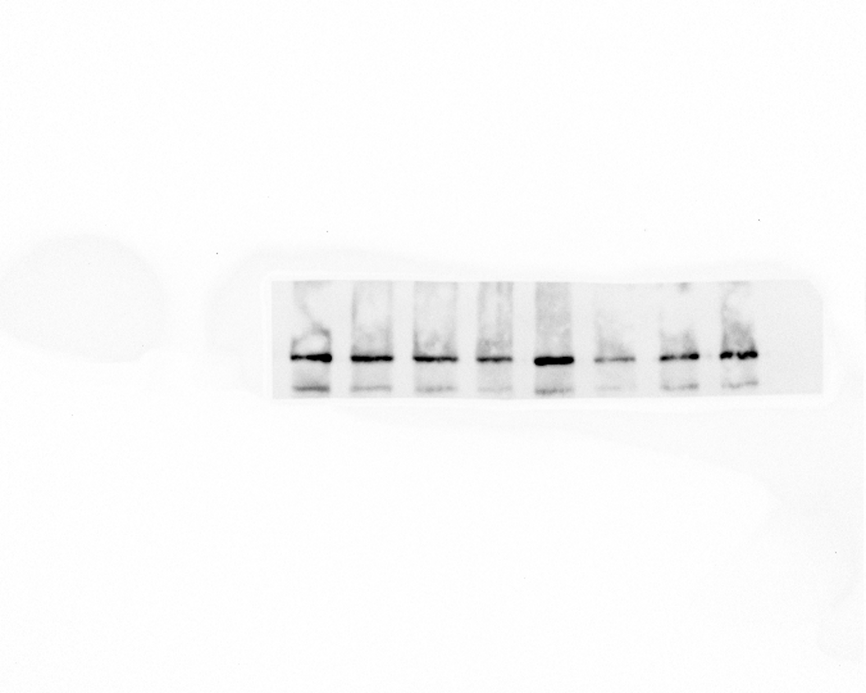


pSTAT3


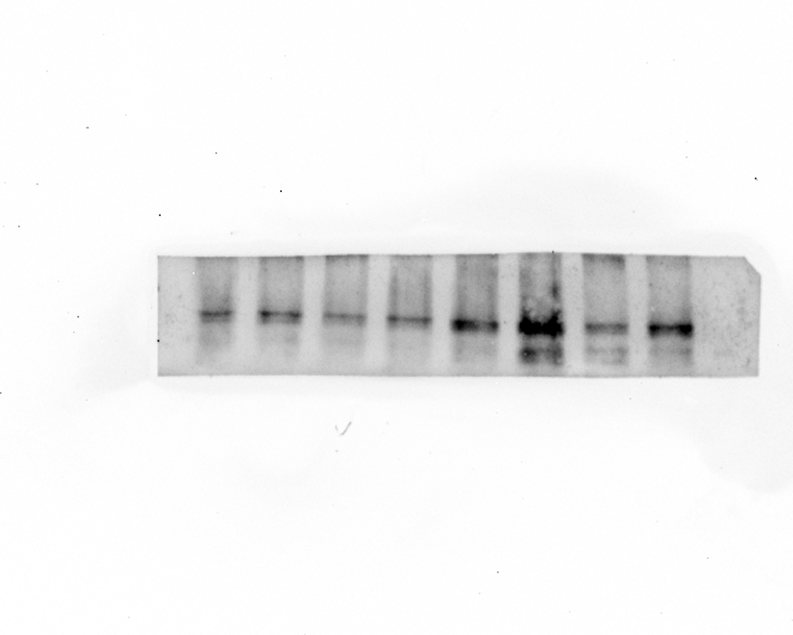


STAT3


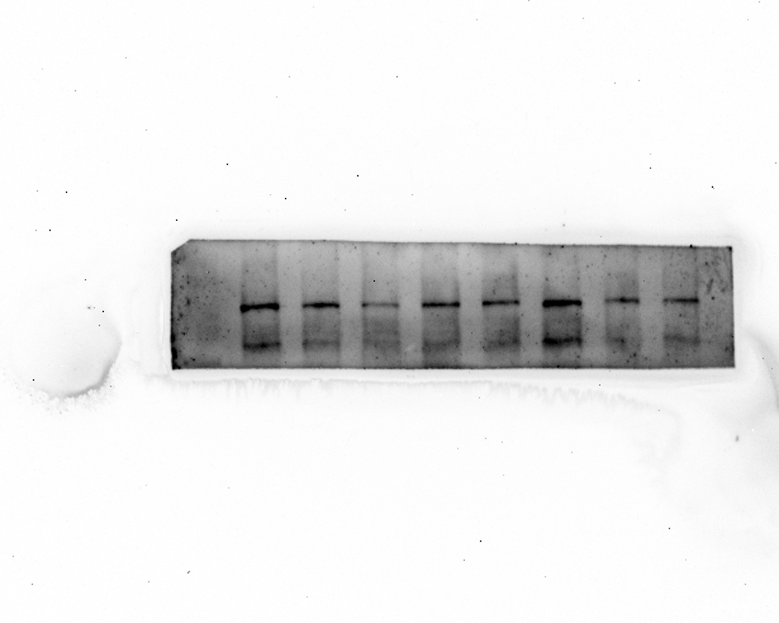


GAPDH


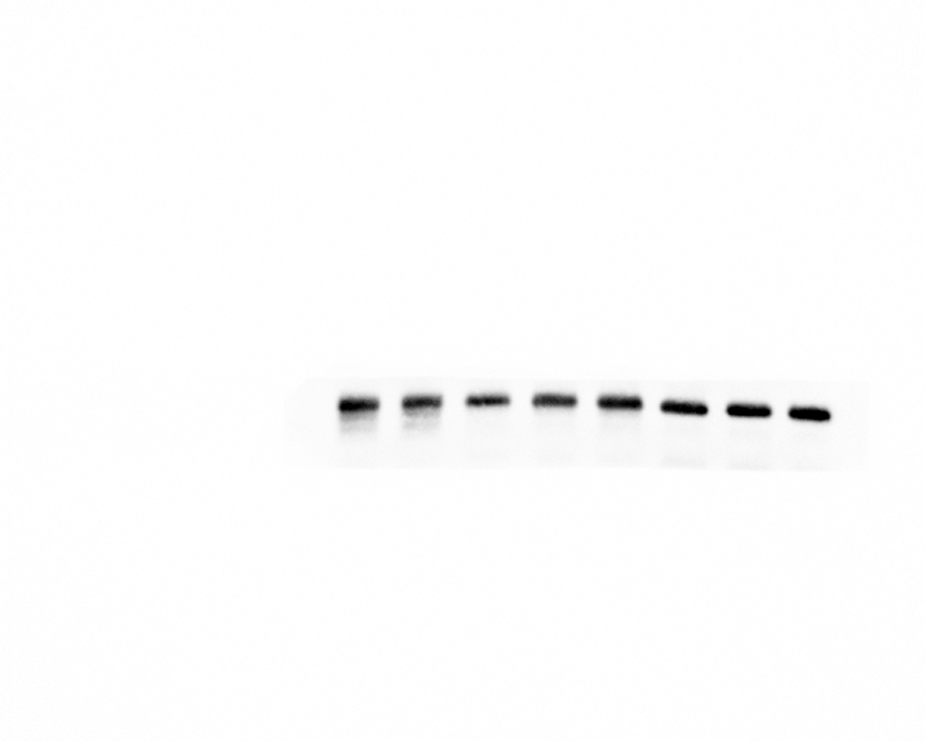


**unprocessed images of WB in Fig 6**

Order:

| PBS | PBS | LM∆ & LI∆ | LM∆ & LI∆ | E6E7 | E6E7 | Others | Others |
| --- | --- | --- | --- | --- | --- | --- | --- |

pJAK1


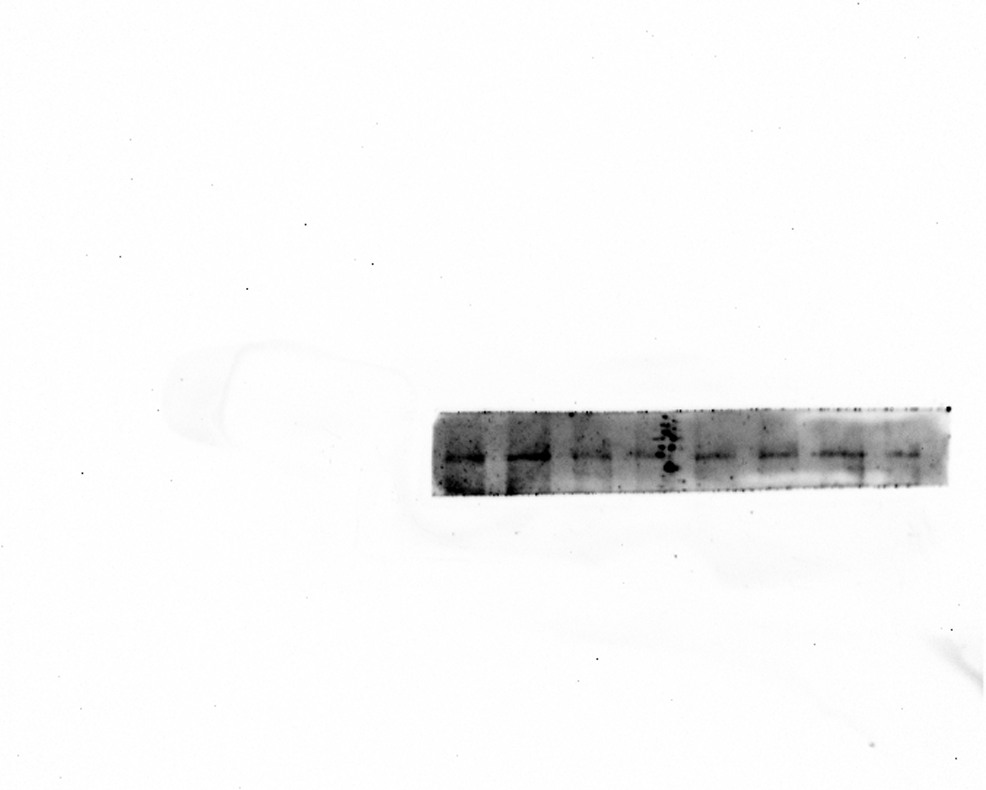


JAK1


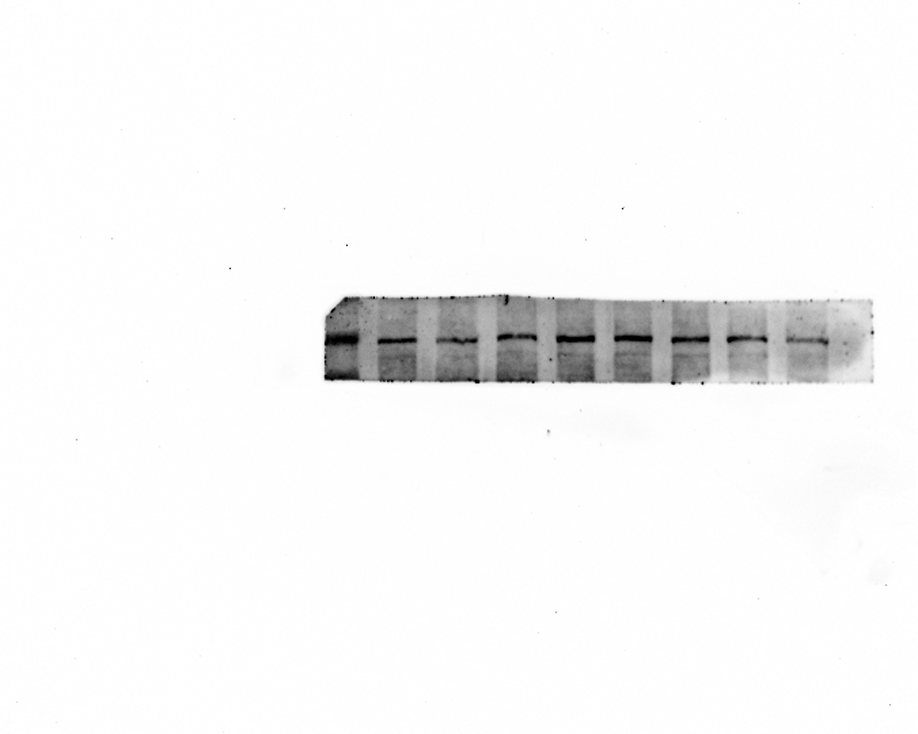


pJAK2


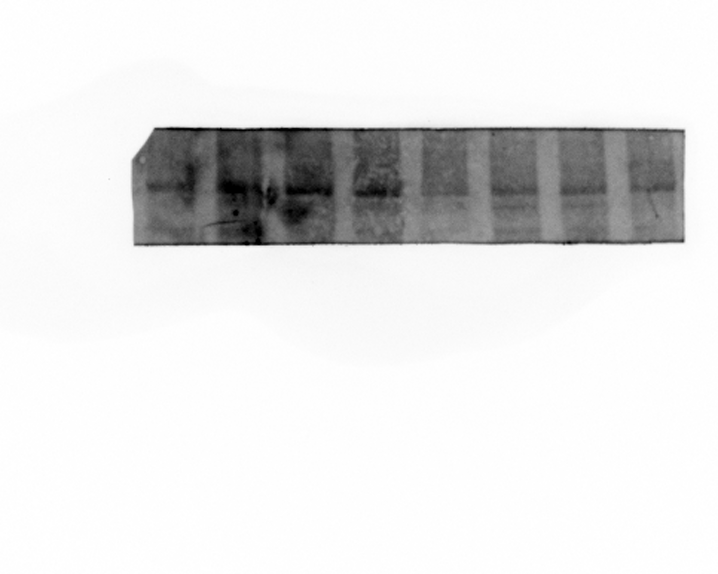


JAK2


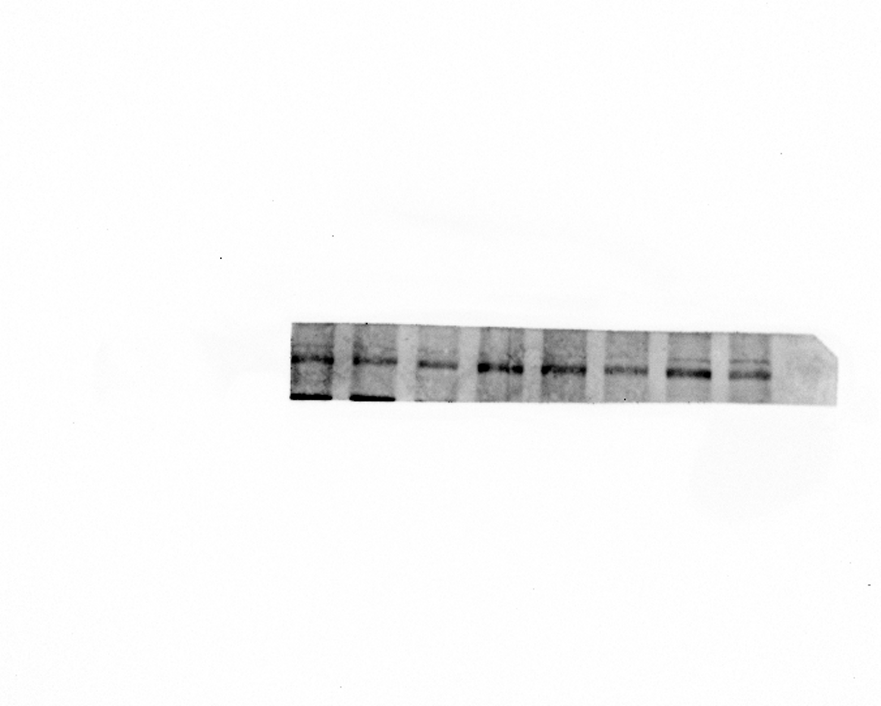


pJAK3


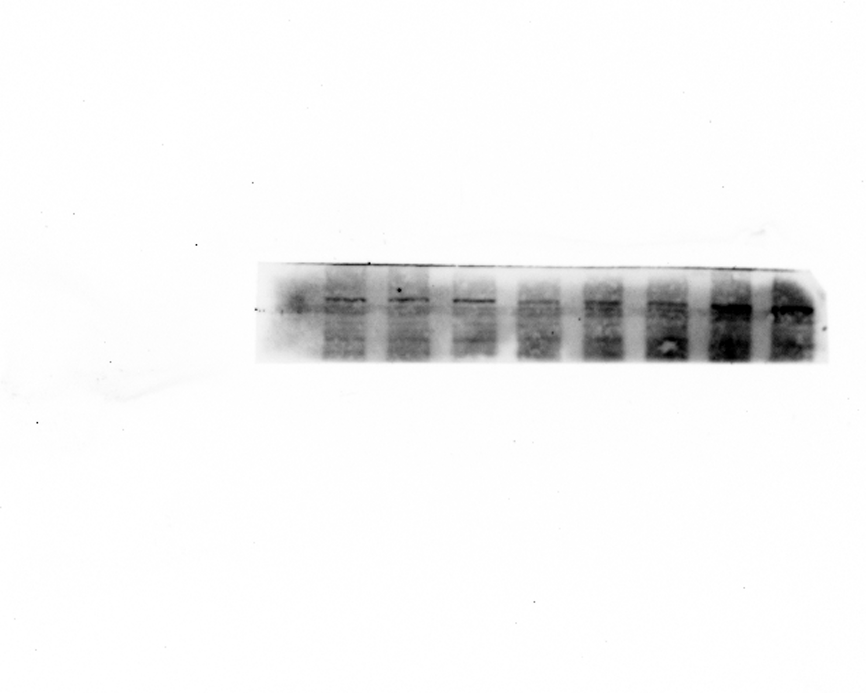


JAK3


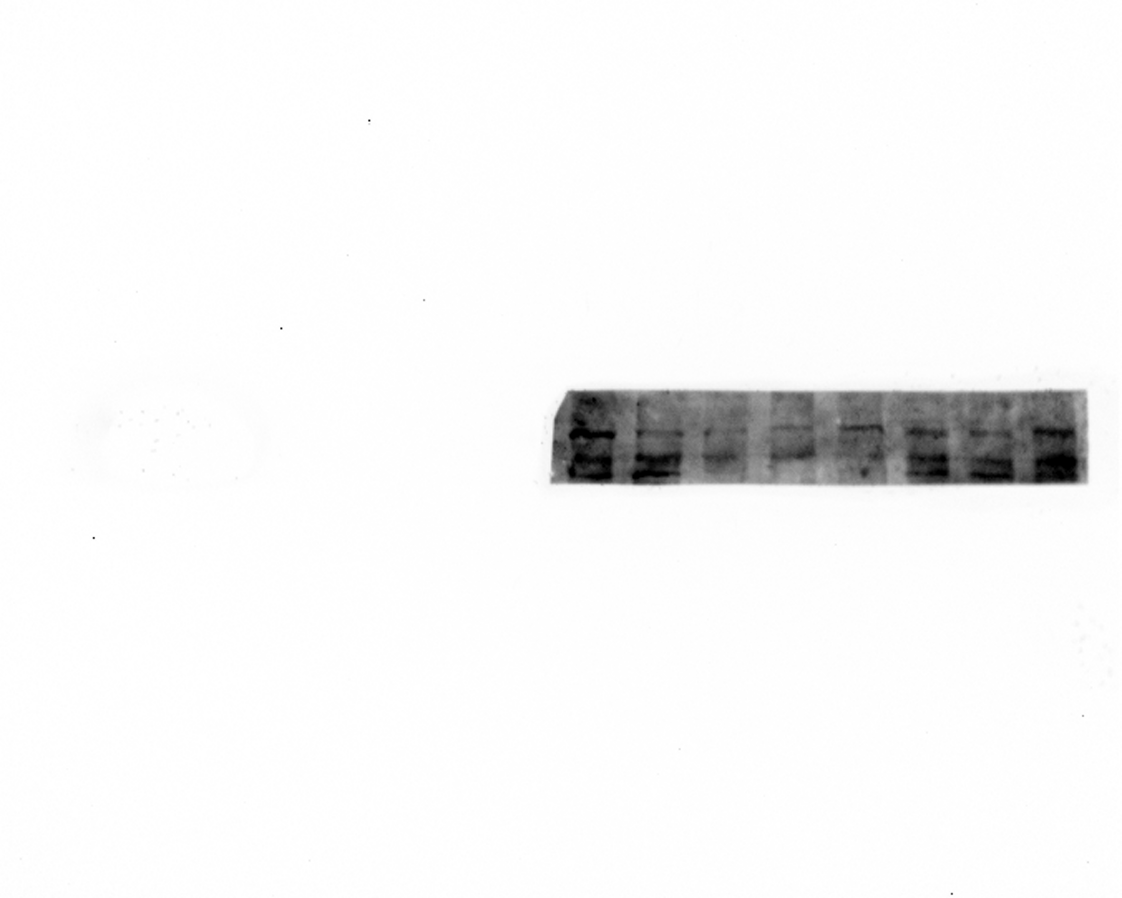


pSTAT1


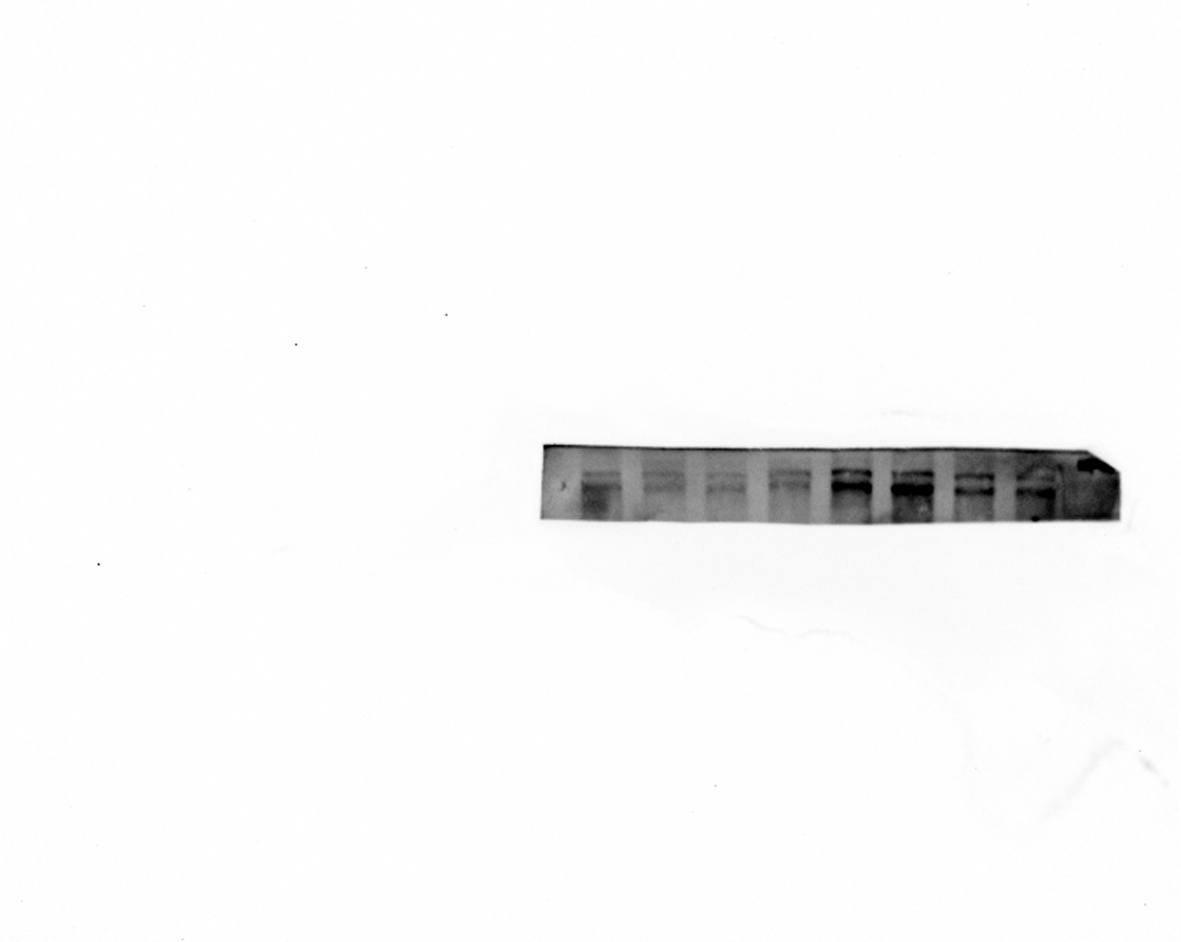


STAT1


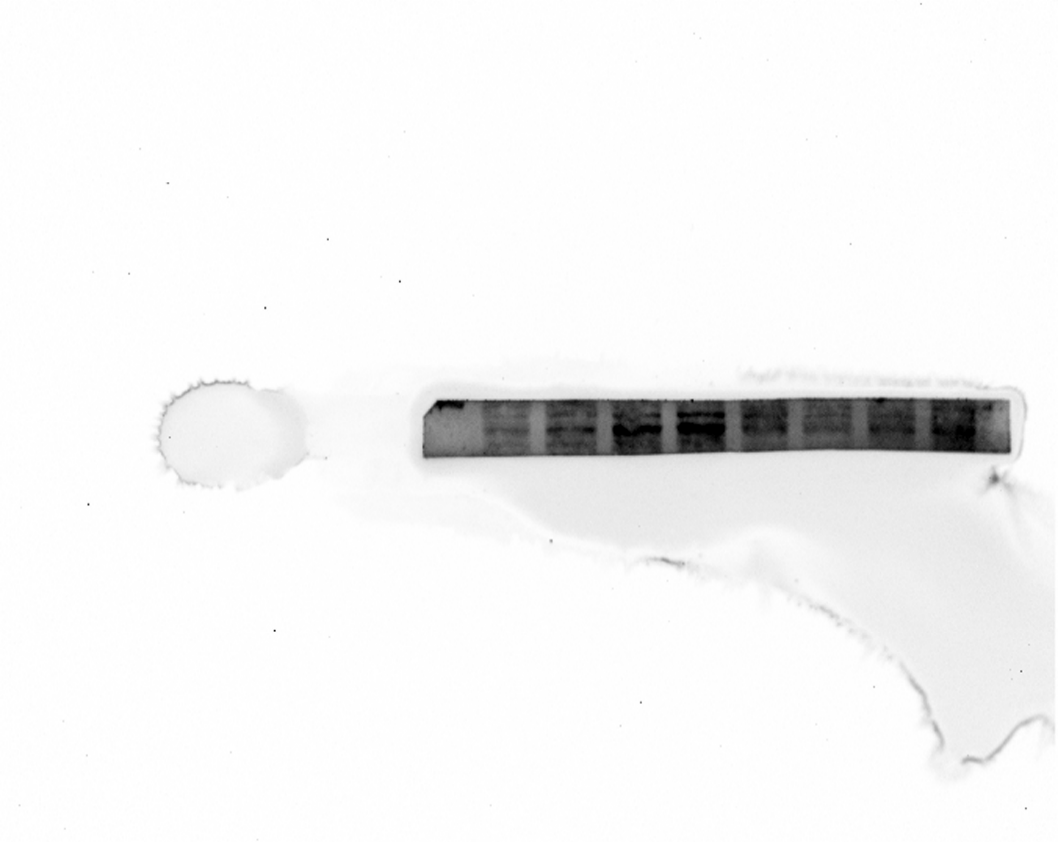


pSTAT3


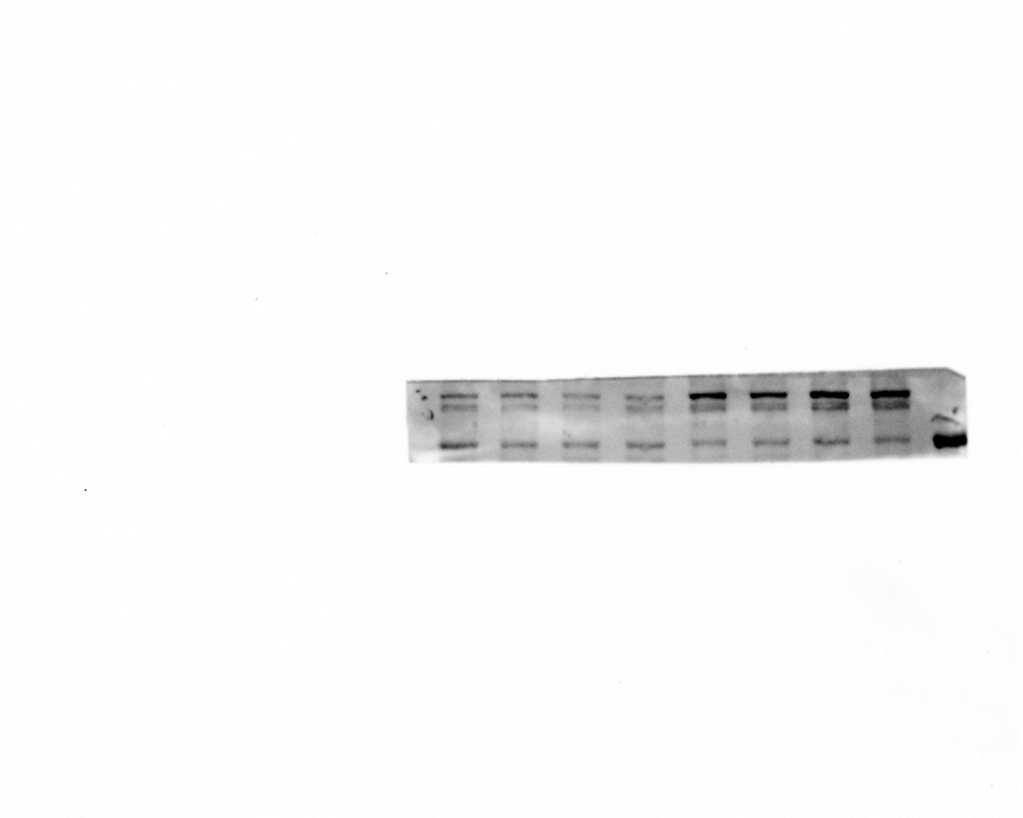


STAT3


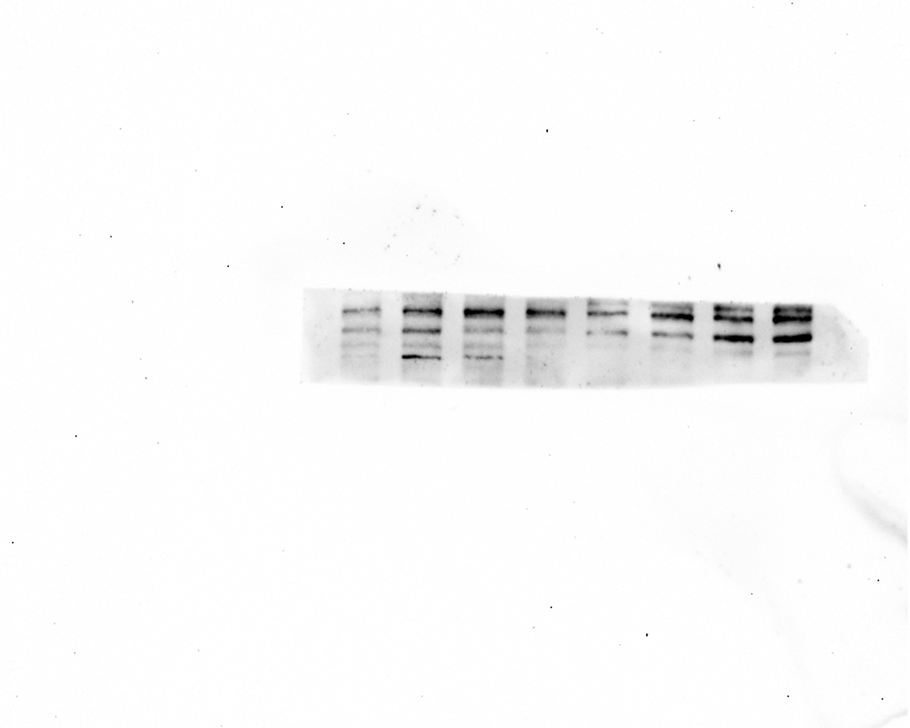


pSTAT5


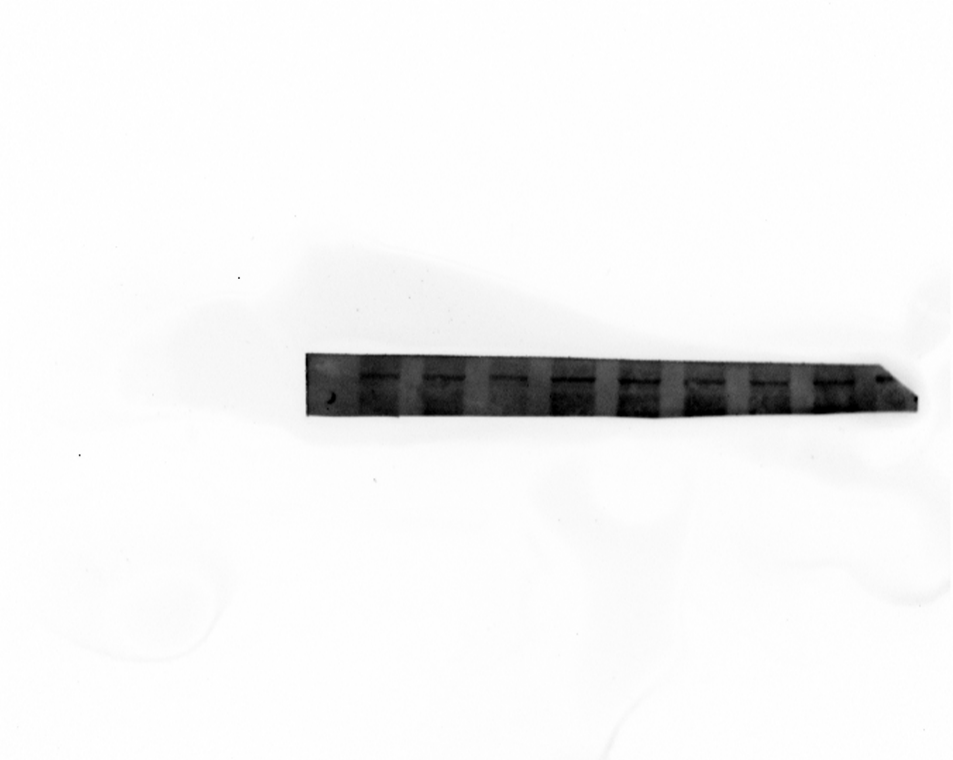


STAT5


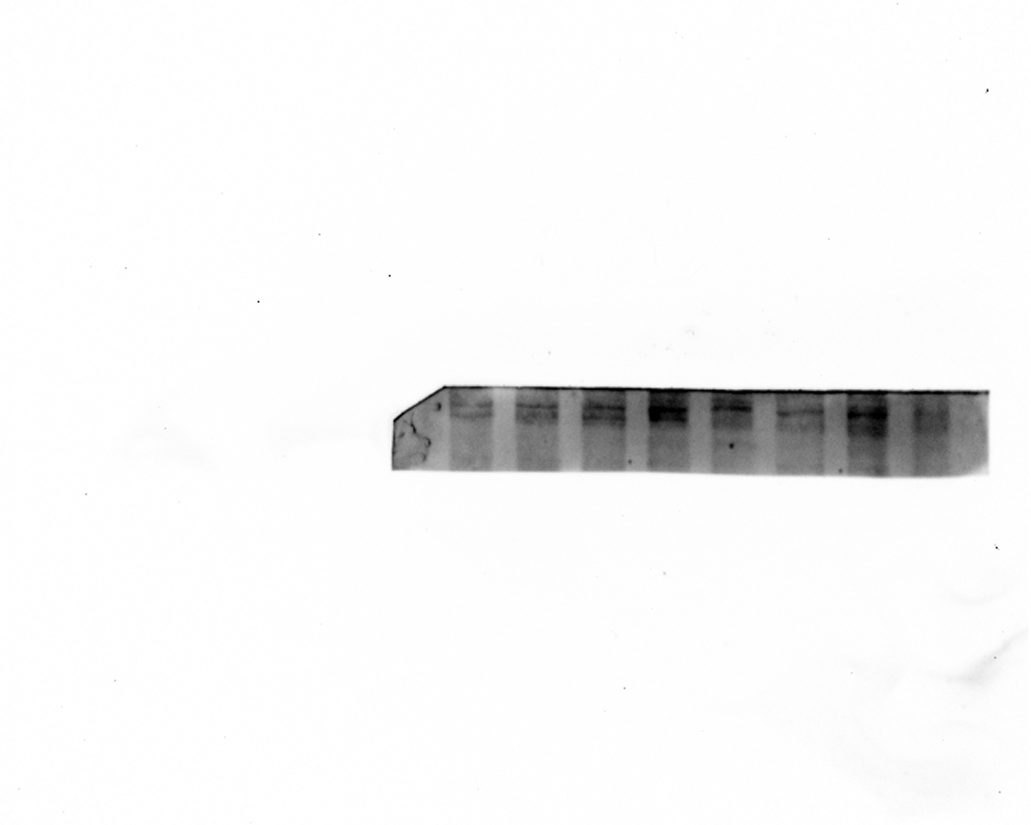


pSTAT6


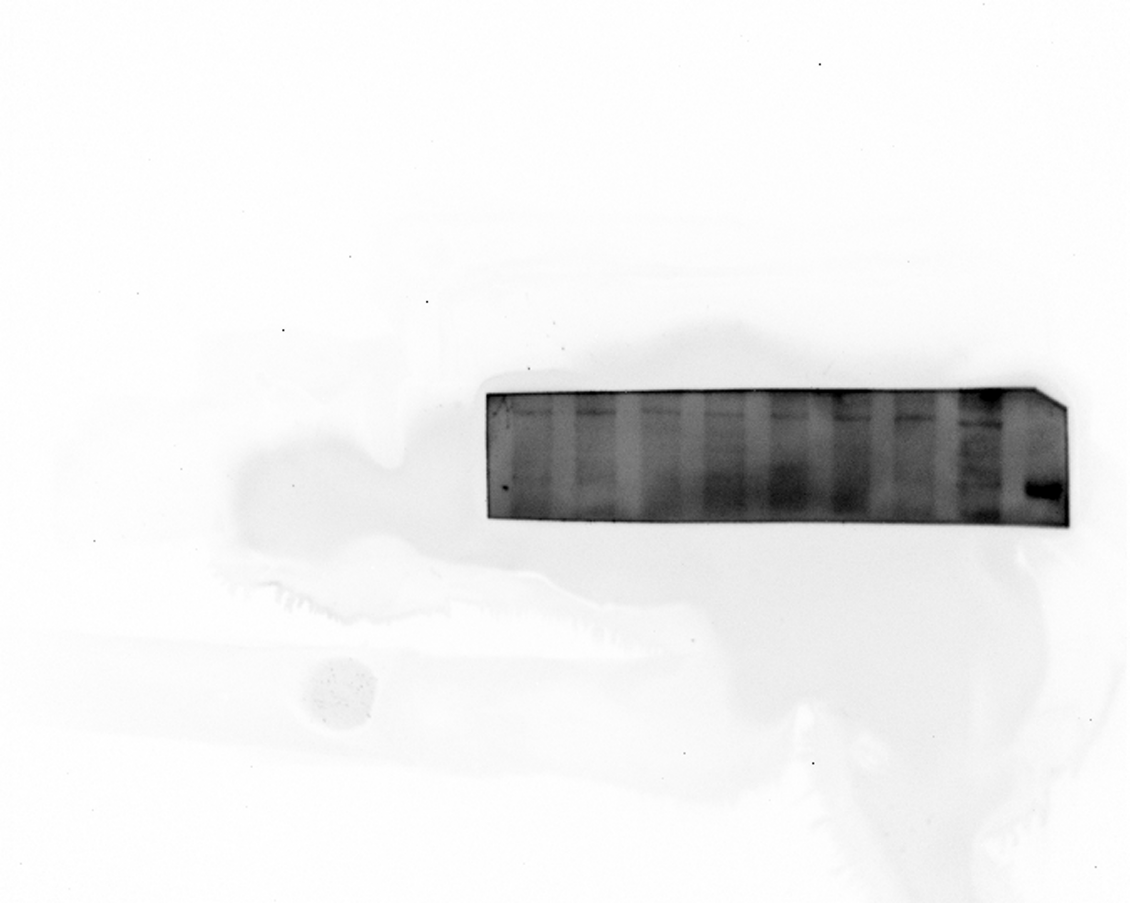


STAT6


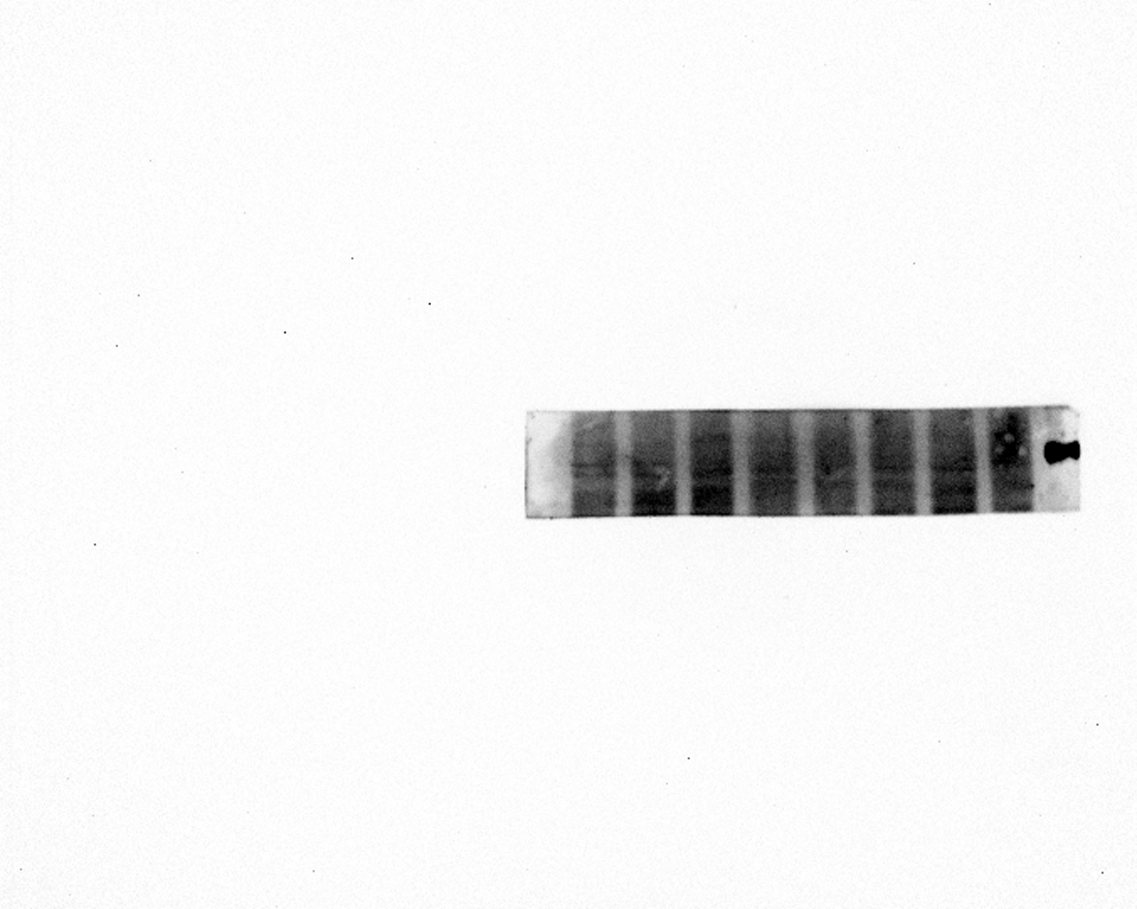


GAPDH


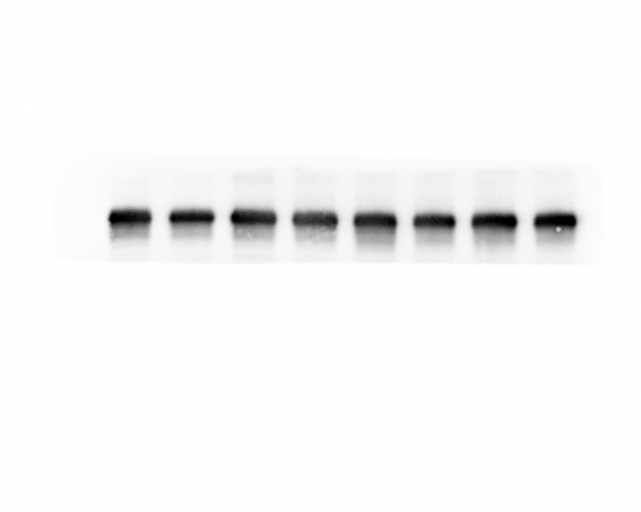


IL-10


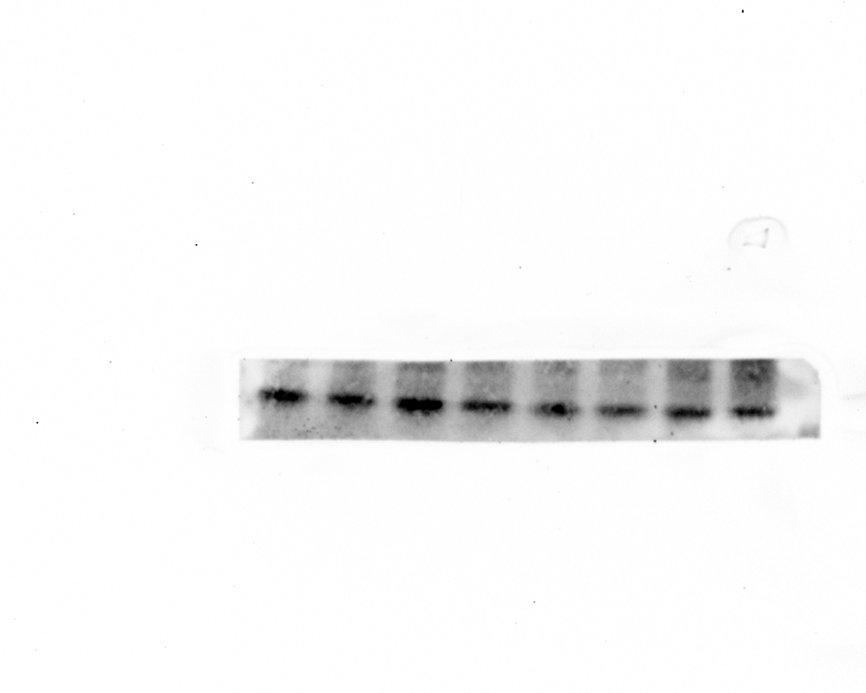


IDO


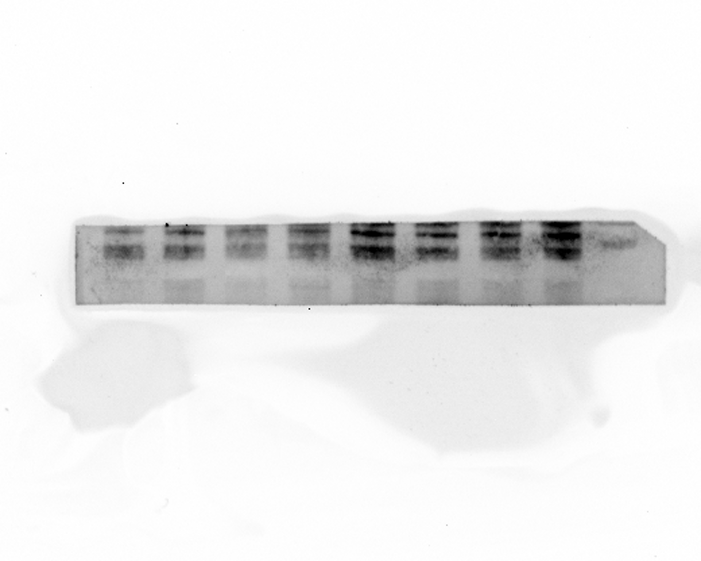


ARG-1


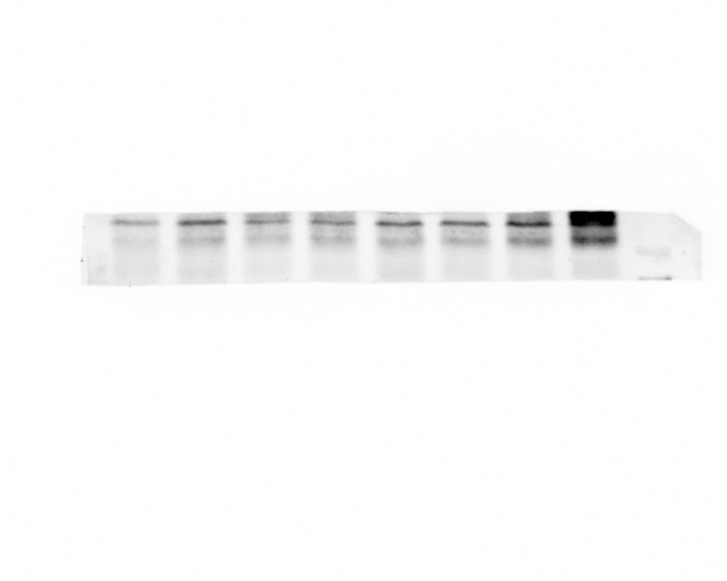


TGF-β1


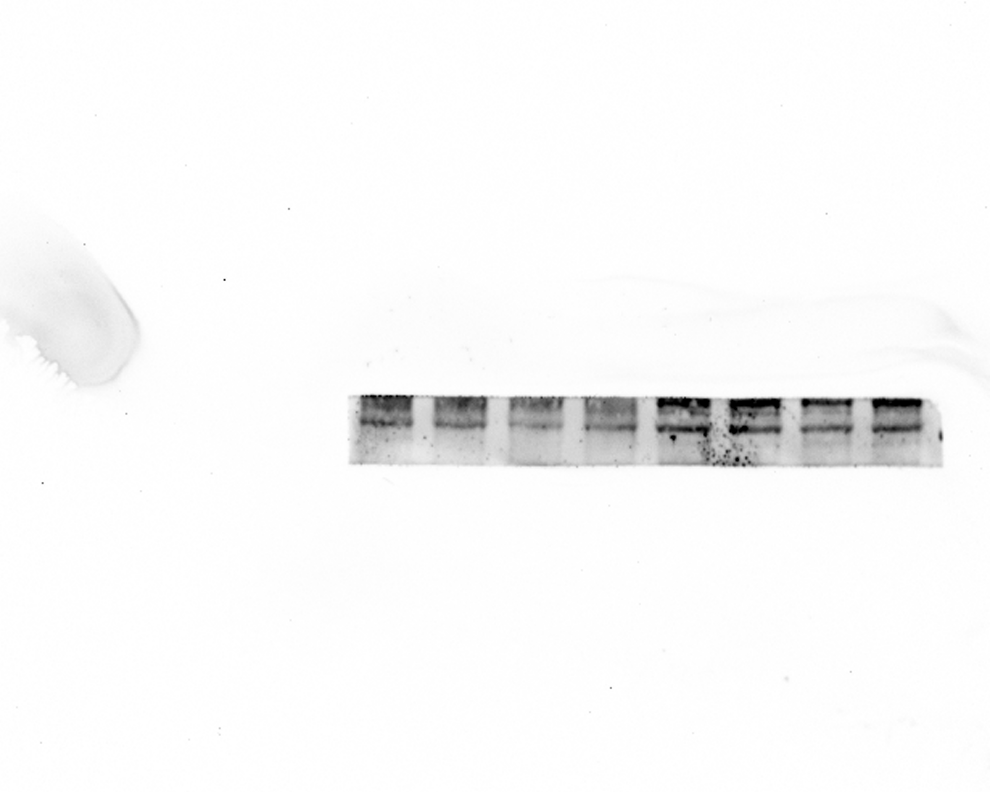


iNOS


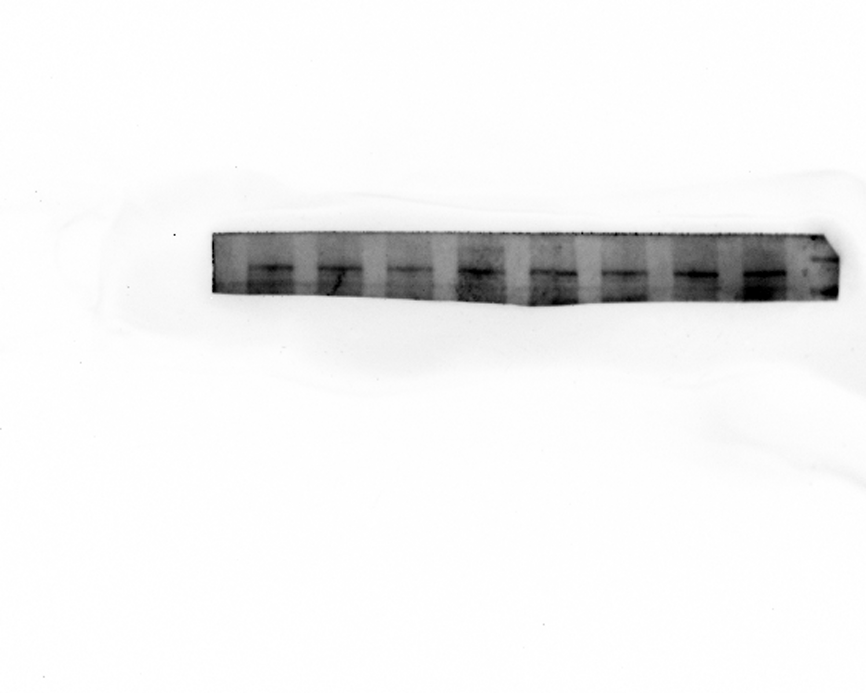


GAPDH


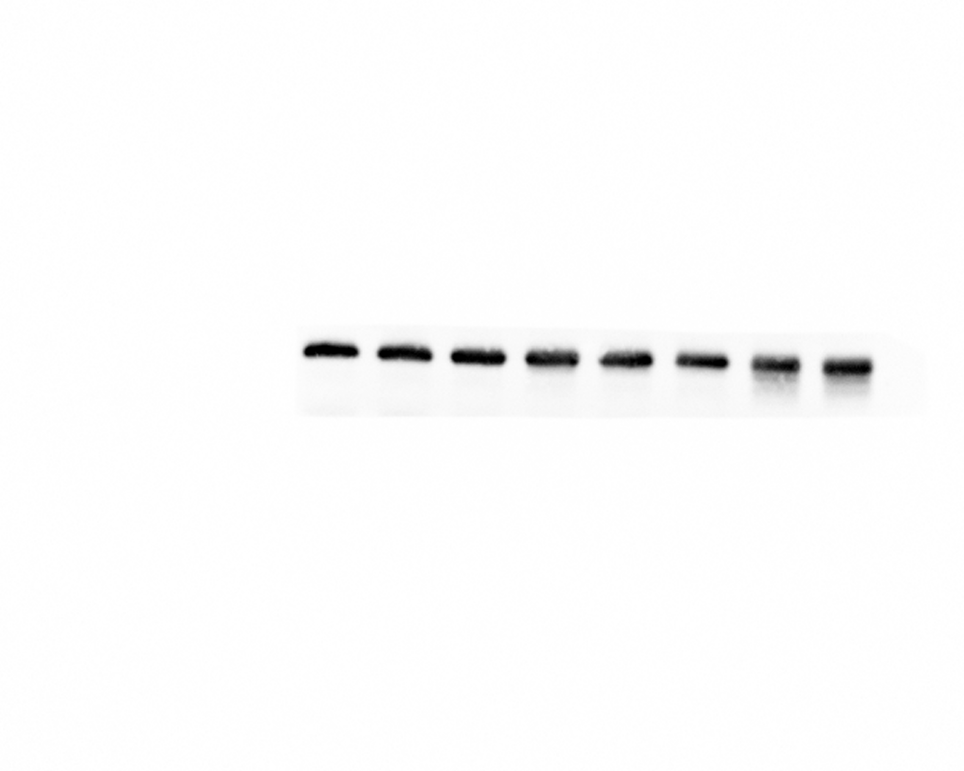

Supplement: Supplementary file 1 — Additional file 1: Table S1 Primer sequence information of genes. Table S2 The concentration and inoculum volume of each strain. Table S3 The information of antibody used in flow cytometry. Table S4 The information of antibody used in WB. Figure S1 Effect of combined immunotherapy with LM∆E6E7 and LI∆E6E7 on mRNA expression levels of downstream genes of JAK-STAT pathway in mice tumor tissues. Figure S2 Gating strategy of flow cytometric analysis of immune cells in the spleen of mice. Figure S3 Gating strategy of flow cytometric analysis of immune cells in the TILs of mice. Figure S4 Gating strategy of flow cytometric analysis of MDSCs in bone marrow cells of mice. The images of the original, uncropped blots. [file 12915_2024_1876_MOESM1_ESM.docx]
